# Mitochondrial nicotinamide adenine dinucleotide reduced (NADH) oxidation links the tricarboxylic acid (TCA) cycle with methionine metabolism and nuclear DNA methylation

**DOI:** 10.1371/journal.pbio.2005707

**Published:** 2018-04-18

**Authors:** Oswaldo A. Lozoya, Inmaculada Martinez-Reyes, Tianyuan Wang, Dagoberto Grenet, Pierre Bushel, Jianying Li, Navdeep Chandel, Richard P. Woychik, Janine H. Santos

**Affiliations:** 1 Genome Integrity and Structural Biology Laboratory, National Institute of Environmental Health Sciences, National Institutes of Health, Durham, North Carolina, United States of America; 2 Department of Medicine, Northwestern University Feinberg School of Medicine, Chicago, Illinois, United States of America; 3 Integrative Bioinformatics Group, National Institute of Environmental Health Sciences, National Institutes of Health, Durham, North Carolina, United States of America; 4 Biostatistics and Computational Biology Group, National Institute of Environmental Health Sciences, National Institutes of Health, Durham, North Carolina, United States of America; University of Washington, United States of America

## Abstract

Mitochondrial function affects many aspects of cellular physiology, and, most recently, its role in epigenetics has been reported. Mechanistically, how mitochondrial function alters DNA methylation patterns in the nucleus remains ill defined. Using a cell culture model of induced mitochondrial DNA (mtDNA) depletion, in this study we show that progressive mitochondrial dysfunction leads to an early transcriptional and metabolic program centered on the metabolism of various amino acids, including those involved in the methionine cycle. We find that this program also increases DNA methylation, which occurs primarily in the genes that are differentially expressed. Maintenance of mitochondrial nicotinamide adenine dinucleotide reduced (NADH) oxidation in the context of mtDNA loss rescues methionine salvage and polyamine synthesis and prevents changes in DNA methylation and gene expression but does not affect serine/folate metabolism or transsulfuration. This work provides a novel mechanistic link between mitochondrial function and epigenetic regulation of gene expression that involves polyamine and methionine metabolism responding to changes in the tricarboxylic acid (TCA) cycle. Given the implications of these findings, future studies across different physiological contexts and in vivo are warranted.

## Author summary

Epigenetic changes in the nucleus play a role in the regulation of gene expression, and metabolism has been shown to affect the epigenome. As mitochondria are not only the powerhouses but also important metabolic hubs in cells, changes in mitochondrial metabolism are likely to affect the epigenome and, as such, gene expression. However, it remains unclear whether mitochondrial function can affect epigenetics, and if such effects alter gene expression. In this work, we used cell culture systems in which mitochondrial function was genetically manipulated to simultaneously study the effects on metabolism, the epigenome, and gene expression. Such approach revealed that loss of mitochondrial respiration leads to a cell-wide metabolic rewiring primarily centered on amino acids, which altered DNA methylation in the nucleus by affecting methionine and polyamine metabolism. We found that these changes preferentially occur in genes responding to mitochondrial dysfunction. Most notably, all these effects could be reversed by maintaining nicotinamide adenine dinucleotide reduced (NADH) oxidation in the mitochondria, revealing a novel link between these organelles and DNA methylation. Given the involvement of mitochondria in many disease conditions, this work opens new challenges and opportunities to understand and potentially manipulate mitochondrial function in health and disease.

## Introduction

Mitochondrial function is key to normal cellular physiology, given the many different biochemical process that occur in the organelle [1]. A tremendous amount of effort over the past several decades has been dedicated to understanding how mitochondrial dysfunction impacts the cellular environment and organismal health. This has been largely based on studies of rare mitochondrial diseases that share many molecular mechanisms with more common disorders that also present with mitochondrial dysfunction, e.g., Parkinson’s disease, cancer, and diabetes. Despite these efforts, fundamental aspects of how mitochondria function impacts cellular physiology remain ill defined. For instance, how the mitochondria communicate with and impact reactions within the nucleus is poorly understood. The gene expression program(s) and metabolic rewiring that change in response to mitochondrial dysfunction are not clear. It is also not known whether mitochondrial-driven epigenetic changes impact gene transcription. Finally, despite the fact that mitochondrial dysfunction can increase DNA methylation [2,3], a mechanistic link between these effects is still missing.

We recently described a novel cell culture model of progressive mitochondrial DNA (mtDNA) depletion in the human embryonic kidney 293 (HEK293) background [4]. This model relies on the inducible expression of a mutant mtDNA polymerase gamma that works as a dominant negative, herein called DN-POLG (dominant-negative DNA polymerase gamma transgene). Upon addition of doxycycline, DN-POLG is expressed, and over a period of 9 days, the mtDNA is completely depleted. Because the mtDNA encodes critical components of the electron transport chain (ETC)—which generates ATP using tricarboxylic acid (TCA) cycle–derived nicotinamide adenine dinucleotide reduced (NADH) or flavin adenine dinucleotide hydroquinone (FADH_2_)—by depleting the mitochondrial genome, we can regulate electron transport, ATP production, and the flux through the TCA cycle. Additionally, because a membrane potential (ΔΨm) is generated as a byproduct of the ETC, producing reactive oxygen species (ROS), we can also modulate redox signaling. Using this model, we showed that complete loss of mtDNA and of ETC function (achieved at day 9) led to severe mitochondrial dysfunction and loss of cell proliferation. We also showed that, concomitant to loss of mtDNA, histone acetylation was decreased in the nucleus. Using isogenic cells that ectopically express 2 nonmammalian proteins—NADH dehydrogenase-like 1 (NDI1) and alternative oxidase (AOX)—we found that maintenance of NADH oxidation with a pseudo-ETC was sufficient to preserve levels of histone acetylation but had no impact on cellular proliferation in the absence of mtDNA. Conversely, by deleting ATPase inhibitory factor subunit 1 (ATPIF1), a regulatory subunit of the mitochondrial ATPase, we showed that maintenance of the ΔΨm without rescue of histone acetylation or ATP production sustained cell proliferation under conditions of complete mtDNA loss, seemingly by restoring redox signaling [4].

The unique progressive nature of mtDNA depletion and mitochondrial dysfunction of this cell culture system provides an exceptional opportunity to fill some of the gaps of knowledge in the field. In this study, we took advantage of this model to gain insights into how cells respond to stepwise mitochondrial dysfunction from transcriptomic, metabolic, and epigenetic perspectives. Our approach revealed that metabolic, epigenetic, and gene expression changes initiate prior to detectable signs of mitochondrial dysfunction, primarily centering on an amino acid response that also aims to sustain the TCA. Unexpectedly, we found that polyamine metabolism is significantly changed upon mitochondrial dysfunction; this, in turn, impacts the methionine cycle and DNA methylation in ways that are independent of serine-driven one-carbon (1C) remodeling or transsulfuration.

## Results

### MtDNA depletion drives dynamic transcriptional changes centered on acetyl coenzyme A (acetyl-CoA) metabolism

The progressive nature of mtDNA depletion in the DN-POLG system provided us with a unique opportunity to address fundamental questions about the nuclear response to progressive mitochondrial dysfunction. We took an integrative approach that involved transcriptomic, metabolomics, and epigenetic analyses at each time point (days 0, 3, 6, and 9) during the course of complete mtDNA depletion. Furthermore, we utilized the cells expressing NDI1/AOX to define the responses to complete mtDNA loss that were specifically linked to NADH oxidation in the mitochondria. A schematic representation of this integrative approach is shown in S1A Fig. The reproducibility of the results is shown in S1B–S1G Fig.

**Fig 1:**
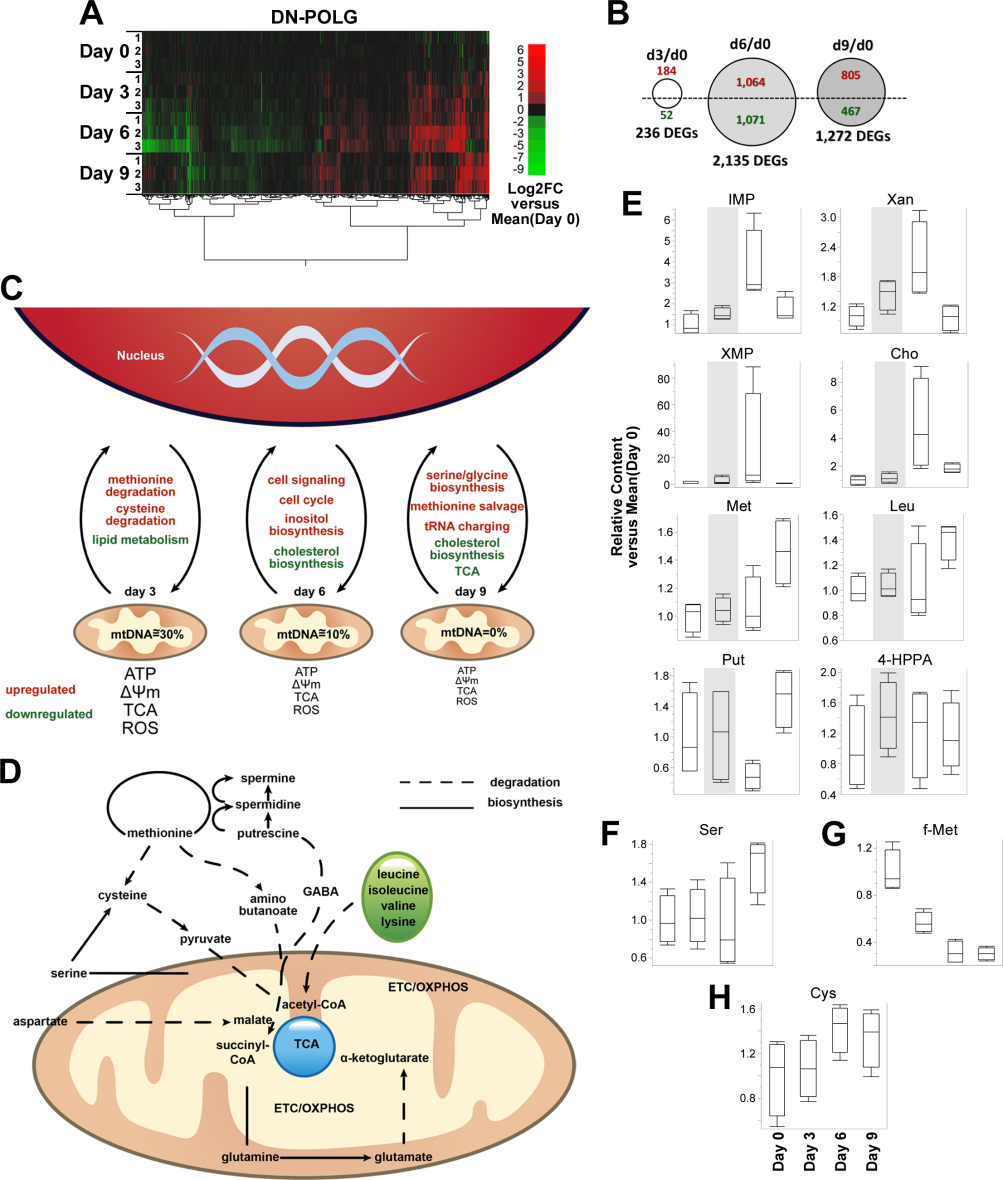
Differential regulation of transcriptional and metabolic profiles in DN-POLG cells in the course of inducible mtDNA depletion. RNA-seq was performed from DN-POLG cells at days 0, 3, 6 and 9 of doxycycline supplementation; *N* = 3 biological replicates per time point. Transcriptional profiling was based on DEGs identified using log2-transformed fold-changes in RPKM versus the mean at day 0 (Log2FC) in two-way ANOVA tests (gene × time) at an adjusted Benjamini-Hochberg *p* ≤ 0.05. (A) Unsupervised clustering analysis of log_2_-fold expression changes versus average of day 0 for DEGs observed in DN-POLG cells by RNA-seq; increased (red) or decreased (green) expression. (B) Number of DEGs identified in DN-POLG cells at days 3, 6 and 9 of doxycycline supplementation compared to day 0 (circle plots, top). (C) Schematic representation of the main transcriptional responses associated to DEGs in DN-POLG cells at days 3, 6 and 9 of dox-inducible mtDNA depletion in DN-POLG cells, per IPA. (D) Schematic representation of interconnected amino acid and methionine metabolic pathways associated to differentially enriched metabolites at days 3, 6, and 9 of dox-inducible mtDNA depletion in DN-POLG cells (*N* = 4 per time point), per IPA. (E–H) Box plots of relative content versus day 0 (IQR-outlier format) depict distribution of individual replicates in DN-POLG cells within statistical groups for (E) IMP, Xan, XMP, Cho, Met, Leu, Put, 4-HPPA, (F) Ser, (G) f-Met, and (H) Cys; partial gray backgrounds highlight metabolite relative contents at day 3 in (E). Underlying data are reported in S1 Data for (A) and (B); S2 Data for (C) and (D); and S3 Data for (E–H). 4-HPPA, 4-hydroxyphenylpyruvate; Cho, choline; Cys, cysteine; DEGS, differentially enriched genes; DN-POLG, dominant-negative DNA polymerase gamma transgene; ETC, electron transport chain; f-Met, N-formylmethionine; GABA, γ-aminobutyric acid; IMP, inosine 5'-monophosphate; IPA, Ingenuity Pathway Analysis; Leu, leucine; Met, methionine; mtDNA, mitochondrial DNA; OXPHOS, oxidative phosphorylation; Put, putrescine; RNA-seq, RNA sequencing; ROS, reactive oxygen species; RPKM, reads per kilobase per million; Ser, serine; TCA, tricarboxylic acid; Xan, xanthine; XMP, xanthosine 5'-monophosphate.

We started by performing RNA sequencing (RNA-seq) at days 0, 3, 6, and 9 in the DN-POLG cells, and found 2,854 genes (S1 Data), including all mtDNA-encoded transcripts, whose expression was changed (adjusted *p* ≤ 0.05) at any given day compared to day 0 (Fig 1A). When stratifying by day, we found 236 differentially expressed genes (DEGs) at day 3, which were mostly (78%) upregulated, while we found 2,135 DEGs at day 6 (1,064 upregulated and 1,071 downregulated). At day 9 we found 1,272 DEGs, most of which (64%) were upregulated (Fig 1B). Common to all time points were 121 DEGs, including POLG that was upregulated at least 7-fold relative to day 0 (S1 Data). The identification of over 200 DEGs at day 3 was surprising given the lack of significant changes in mitochondrial function at this time [4]. Our model also revealed progressive upregulation of fibroblast growth factor 21 (FGF21) and growth differentiation factor 15 (GDF15) relative to day 0 (S1 Data). FGF21 and GDF15 are metabolic cytokines induced in patients and mouse models of mtDNA or protein translation defects, which have been proposed as biomarkers of mitochondrial dysfunction [1]. Validation of randomly selected genes by quantitative real-time PCR can be found in S2A Fig.

The use of Ingenuity Pathway Analysis (IPA) revealed that, globally, the genes modulated during the course of mtDNA depletion enriched for tRNA charging, cholesterol biosynthesis, glutamate (via 4-aminobutyrate) and putrescine degradation, and D-myo-inositol-5-phosphate metabolism (top 10, S2 Data). These results are consistent with the loss of mtDNA having a negative impact on oxidative TCA flux, since glutamate degradation can contribute to the cycle as precursor for succinate. Likewise, putrescine can contribute to the TCA as succinate via γ-aminobutyric acid (GABA) catabolism [5], known to occur in cells beyond the central nervous system [6]. To our knowledge, this is the first report to identify polyamine metabolism as responsive to loss of mitochondrial function. Inositols are sensitive to glucose and NADH levels [7], the metabolism of which is also impacted by loss of mtDNA. To understand how DN-POLG cells respond to progressive loss of mtDNA, we next stratified the analysis based on time point and the directionality of gene changes. We reasoned that the genes modulated at day 3 would reflect early responses to mtDNA depletion that take place prior to signs of mitochondrial dysfunction [4]. Those DEGs at day 6 would reveal responses to significant loss of ETC function and the pathways directly linked to this process—e.g., ATP production and the oxidative TCA—while the ones detected at day 9 would reveal adaptive changes within the cell. A summary of the main transcriptional responses identified at each time point is shown in Fig 1C.

Most genes detected at day 3 (184) were upregulated and enriched for methionine and cysteine degradation (S2 Data). Alterations in the methionine cycle have not been directly associated with mitochondrial dysfunction, although channeling of 1C units toward transsulfuration of homocysteine to cysteine, a branch point in the methionine cycle, has been recently reported [8,9]. However, the degradation of methionine can ultimately input into the TCA cycle by contributing pyruvate (through cysteine) and succinyl-CoA through aminobutanoate [10–12]; this would provide a link between mtDNA depletion and methionine degradation. Nevertheless, it was surprising to identify these changes at day 3 when no significant alterations in mitochondrial function were identified [4]. Concomitant to the degradation of methionine, we observed DEGs involved in the recycling (or salvage) of this amino acid through betaine, which was likely an attempt to maintain methionine levels (S1 and S2 Data). The 52 genes downregulated at day 3 were enriched for lipid metabolism, presumably to spare acetyl-CoA, and endothelial nitric oxide synthase (eNOS) signaling (S2 Data).

The 2,135 DEGs identified at day 6 enriched for pathways involved in cell signaling, cell cycle regulation, and inositol metabolism, which were driven by the upregulated genes (S2 Data). The 1,071 downregulated genes enriched primarily for inhibition of cholesterol biosynthesis, which is in line with the suppression of fat metabolism initiated at day 3 (S2 Data). Because cell proliferation is affected at day 6 [4] and cholesterol has roles in membrane structures, inhibition of cholesterol biosynthesis—in addition to sparing acetyl-CoA—may be a response to loss of cell division.

When mtDNA was fully depleted at day 9, the 805 upregulated genes enriched for serine and glycine metabolism, as was recently reported by others [8,9], and for methionine salvage through betaine. The use of betaine to recycle methionine, which is a folate-independent pathway, may reflect serine-associated folate being channeled to purine metabolism [9]. We also identified changes in tRNA charging, which suggests an attempt by the cell to preserve cytosolic protein synthesis (S2 Data). Inhibition of cholesterol biosynthesis, as found at day 6, was the top category identified with the 467 DEGs that were downregulated (S2 Data). We found at day 9 that the degradation of several proteinogenic amino acids was inhibited and that the TCA cycle was suppressed (S2 Data); this is consistent with their utilization for protein synthesis rather than, for instance, supplying precursors for the TCA.

IPA can also predict upstream regulators involved in driving the transcriptional programs identified. Activating transcription factor 4 (ATF4) is a transcription factor recently linked to a mitochondrial stress response [13] and was predicted only when evaluating the genes that were upregulated under our experimental conditions, irrespective of the degree of mitochondrial dysfunction (S2B Fig). Conversely, several upstream regulators were predicted to be associated with the downregulated genes, including tumor protein p53 (TP53), MYC, and peroxisome proliferator activated receptor alpha (PPARα). The only gene consistently predicted to play a role in the inhibitory responses at all times was the major facilitator superfamily domain-containing protein 2a (MFSD2A) (S2B Fig), which has been recently linked to fatty acid oxidation [14].

### Changes in purine nucleotides, the methionine cycle, and the TCA drive the early metabolic response to mtDNA depletion

We previously performed a metabolomics analysis in DN-POLG cells at days 0, 3, 6, and 9 and showed that many metabolites were changed during mtDNA depletion [4]. To gain more insights into the progressive remodeling of the metabolome as a function of mtDNA depletion, and to explore the relationship with the transcriptome changes, we used the metabolite data to identify the pathways that were enriched over time. We started by determining those metabolites that were statistically different at any given point relative to day 0, using adjusted *p* ≤ 0.05 and an effect size of 1.15-fold (for more information, see Methods). We found a total of 459 metabolites using these statistical criteria, of which 231 were significantly different at day 3, 396 at day 6, and 345 at day 9 (S3A Fig and S3 Data); common to all time points were 179 metabolites (S3A Fig and S3 Data). We then performed pathway enrichment analysis using the 459 metabolites, which revealed the dynamic nature of the metabolic changes over time. For example, most pathways progressively enriched between days 3–9, while some initiated at day 6, and others decreased by day 9 (S3B Fig).

The top enriched pathways involved purine nucleotides and the superpathway of methionine degradation, which was also the most significantly enriched pathway across the experimental time course (S3B Fig). The fact that methionine degradation was captured at the transcriptional level already at day 3 (S1 and S2 Data) and was also the highest significant metabolic pathway engaged over time revealed an unexpected connection between methionine metabolism and loss of mtDNA. This finding was consistent with the overall amino acid response identified from the transcriptome data. The metabolite analysis showed the engagement of both catabolic and biosynthetic amino acid pathways; a summary of the main pathways is schematically represented in Fig 1D. Many of enriched pathways for amino acid degradation involved those that can input into the TCA to make acetyl-CoA, like leucine, valine and lysine, or other intermediates such as malate, succinyl-CoA, or α-ketoglutarate (Figs 1D and S3B). Biosynthesis of other amino acids—such as serine, cysteine, and glutamate—was also observed (Figs 1D and S3B). Consistent with amino acid degradation, the urea cycle that recycles ammonia derived from amino acid catabolism was enriched; linked to it was the biosynthesis of citrulline (S3B Fig). The degradation of putrescine, which can input into the TCA as succinate, was also identified (S3B Fig); this was in line with the transcriptome data (S2 Data). It is noteworthy that the urea cycle provides ornithine, the precursor of putrescine, thus offering a constant supply of these metabolites in the DN-POLG cells. The urea cycle, while mostly connected with the liver, occurs partially in the kidneys [15]. Several (although not all) genes involved in this pathway are expressed in different tissues [16,17]. The identification of the urea cycle as enriched in HEK293 cells is likely a reflection of activation of components of the pathway to recycle ammonia, rather than the canonical liver urea cycle, under our experimental conditions. Also, increased degradation of choline—the precursor of betaine—and glycine/betaine metabolism were enriched (S2 Data), which is in agreement with the RNA-seq findings that suggested that methionine levels were maintained through salvage pathways. Various examples of the relationship between the transcriptome and metabolic remodeling can be found in S3C–S3G Fig.

We assumed that the changes found at day 3 would reveal the drivers of the global metabolic response to mtDNA depletion. To define those drivers, we ranked the relevance of the pathways based on the ones most significantly enriched at day 3, focusing arbitrarily only on the ones with a *p* ≤ 10^−7^. What we found were 3 main nodes that essentially centered around purines, the TCA, and redox reactions (S3H Fig). The levels of some metabolites involved in these pathways are shown in Fig 1E. These data suggest, despite the lack of detectable changes in mitochondrial function, that the level of mtDNA depletion achieved at day 3 remodels metabolism in a way that prepares the cells to adjust nucleic acid metabolism (transcription, DNA repair, and replication), cell cycle, protein translation, methylation reactions, and redox homeostasis. This analysis also revealed 6 pathways that were not significantly enriched at day 3 but that were identified at later time points (S3B Fig). These pathways were associated with overt mitochondrial dysfunction and included pyrimidine ribonucleotide interconversion, biosynthesis of cysteine, glutathione, glutamine, and the polyamines spermidine and spermine (S3B Fig).

It was surprising that the biosynthesis of cysteine and glutathione was not engaged at day 3, since mtDNA depletion was recently shown to induce serine biosynthesis (also shown here, at day 3 *p =* 10^−2^; S3B Fig and Fig 1F), channeling 1C metabolism to cysteine and glutathione production through transsulfuration [8,9]. It is worth noting that serine is also involved in the formation of formyl-methionine by feeding into the mitochondrial folate cycle [1]. Formyl-methionine is the unique amino acid used to initiate translation of mtDNA-encoded proteins [18]. Despite significant loss of mtDNA at day 3 (S4A Fig), levels of mtRNA transcripts were stable (S4B Fig), and mtDNA-encoded proteins were not significantly affected [4]. Thus, we hypothesized that serine biosynthesis at day 3 serves to maintain mitochondrial protein translation and sustain organellar function; at later time points, it likely supports cysteine and glutathione production, as shown by others [8,9]. In agreement with this hypothesis, levels of formyl-methionine were higher at day 3 compared to days 6 or 9 (Fig 1G), whereas that of cysteine followed the opposite trend (Fig 1H). The reason why the serine biosynthetic pathway is activated upon mtDNA depletion remains unclear.

### Loss of mtDNA leads to DNA hypermethylation through increased SAM

**Fig 2:**
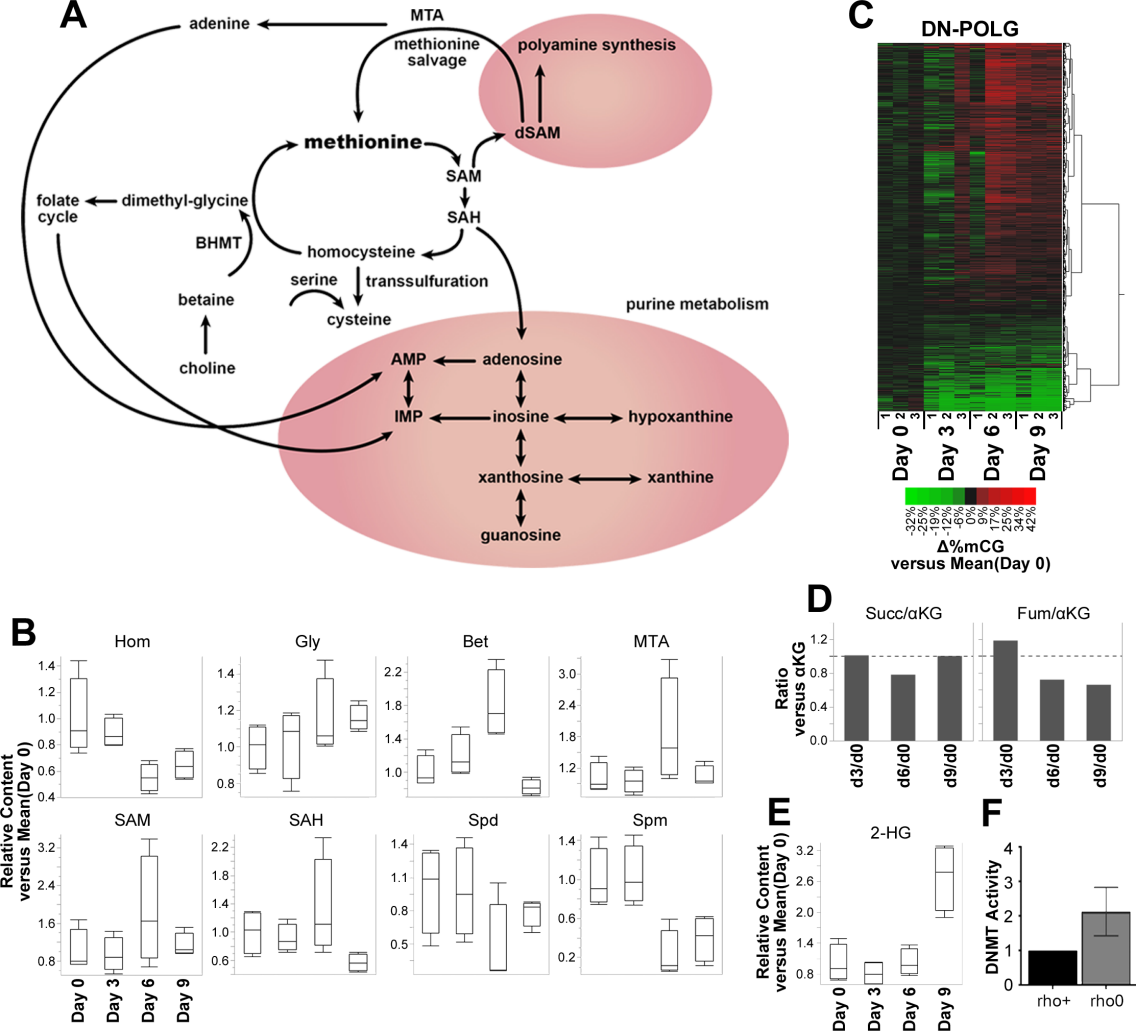
Metabolic response to mitochondrial dysfunction also affects DNA methylation patterns in DN-POLG cells. Metabolomics was performed in DN-POLG cells at days 0, 3, 6 and 9; *N* = 4 per time point. Differentially enriched metabolites were identified based on log2-transformed fold-changes in arbitrary detection units versus the mean at day 0 (y-axis) in each time point during doxycycline treatment (x-axis) by a two-way ANOVA test (metabolite × time) at an adjusted Benjamini-Hochberg *p* ≤ 0.05. (A) Schematic representation of interconnected polyamine synthesis, purine metabolism and methionine salvage pathways associated to differentially enriched metabolites at days 3, 6 and 9 of dox-inducible mtDNA depletion in DN-POLG cells, per Ingenuity Pathway Analysis. (B, E) Box plots of relative content versus day 0 (IQR-outlier format) depict distribution of individual replicates in DN-POLG cells within statistical groups for (B) Hom, Gly, Bet, MTA, SAM, SAH, Spd, Spm, and (E) 2-HG. (C) Unsupervised clustering analysis of Δ%mCG for DML observed in DN-POLG cells by HM-450K BeadArrays; hypermethylation (red) or hypomethylation (green). *N* = 3 per time point. DML were identified based methylation beta-values versus the mean at day 0 (Log2FC) by a two-way ANOVA test (probe × time) at an adjusted Benjamini-Hochberg *p* ≤ 0.05. (D) Average content ratios for Succ and Fum to αKG at days 3, 6, and 9 in DN-POLG cells normalized to the mean at day 0, based on metabolomics output (*N* = 4 per time point). (F) DNMT activity was gauged in 143B rho0 and rho+ cells by following the transfer of radiolabeled methylated substrate onto poly-dIC oligonucleotide. *N* = 3 per cell model; data are presented relative to detected activity in rho+ cells (bar plot: mean ± SEM). Underlying data are reported in S3 Data for (B), (D), and (E); S4 Data for (C); and S9 Data for (F). 2-HG, 2-hydroxyglutarate; αKG, α-ketoglutarate; AMP, adenosine monophosphate; Bet, betaine; BHMT, betaine homocysteine-methyltransferase; Δ%mCG, DNA methylation changes versus average of day 0; DML, differentially methylated genomic loci; DNMT, DNA methyltransferase; DN-POLG, dominant-negative DNA polymerase gamma transgene; Fum, fumarate; Gly, glycine; Hom, homocysteine; IMP, inosine 5'-monophosphate; MTA, 5-methylthioadenosine; mtDNA, mitochondrial DNA; poly-dIC, poly DNA inosinic-polycytidylic acid; SAH, S-adenosylhomocysteine; SAM, S-adenosylmethionine; Spd, spermidine; Spm, spermine; Succ, succinate.

Carbon units derived from folate-1C metabolism are used for the synthesis of purines and the generation of S-adenosyl-methionine (SAM), which is considered the universal methyl donor for DNA, RNA, lipids, and proteins [19,20]. Levels of SAM are also influenced by polyamine synthesis, which uses decarboxylated SAM for the production of spermidine and spermine from putrescine, generating 5-methyl-thioadenosine (MTA). MTA is recycled back into the methionine cycle through a salvage pathway that also produces adenine, thus feeding into the purine pool [21]. Interestingly, MTA has been shown to be the major source of de novo adenine in human cells [22]. Our transcriptomic and metabolic data suggest that the progressive mtDNA depletion achieved over 9 days significantly affects the methionine cycle in various ways, including (i) by channeling homocysteine to transsulforation, (ii) by increasing the utilization of betaine as a folate-independent methionine precursor, (iii) by promoting the degradation of methionine, and (iv) by altering polyamine synthesis and degradation that, in turn, affects MTA recycling (Fig 2A). However, whether these changes impact the levels of SAM, influencing methylation reactions, remains unknown.

We examined the metabolites associated with the methionine cycle (Fig 2A) and found that while homocysteine levels decreased over time (Fig 2B), levels of methionine (Fig 1E), serine (Fig 1F), glycine (Fig 2B), and cysteine (Fig 1H) increased. Choline (Fig 2B), betaine, SAM, and MTA levels were maximal at day 6, returning to levels closer to basal at day 9 (Fig 2B). Levels of S-adenosyl-homocysteine (SAH), the byproduct of SAM metabolism, increased at day 6 and decreased at day 9 below basal levels (Fig 2B). A high SAM/SAH ratio is favorable to methylation reactions since SAH inhibits the methyltransferases [10,23]. Steady state levels of the polyamines putrescine, spermidine, and spermine followed an interesting trend. While putrescine decreased by day 6 and increased by day 9 (Fig 1E), the levels of spermidine and spermine decreased over time (Fig 2B). This effect on the steady state levels of the polyamines is also reflective of an increased catabolism of spermine and spermidine through spermine/spermidine N-acetyl-transferase (SAT1), which is upregulated at the transcriptional level in the DN-POLG (S1 Data) and whose net product is putrescine [24].

Since previous observations that DNA methylation is influenced by mtDNA depletion and mitochondrial dysfunction in cultured cells and animal models [2,3], we hypothesized that the changes in SAM we observed could drive this effect. Specifically, we predicted that the DNA would be hypermethylated, with maximal levels at day 6. To test this hypothesis, we evaluated whole genome DNA methylation status at a single nucleotide resolution using the Illumina 450K platform. We found that mtDNA depletion progressively increased DNA methylation in promoters, gene bodies, or intergenic regions (S5A Fig), with hypermethylation peaking at day 6 and decreasing at day 9 (Fig 2C). Although the changes we detected were somewhat modest (full range of Δ%mCG: −30% to +40%; see Fig 2C) we reasoned they reflected the short time frame of the experiments. Indeed, when evaluating DNA methylation using the same approach in cells chronically depleted of mtDNA (rho0) in the 143B background, we found that methylation changes were more prominent, ranging between −60% and +60% with respect to cells with endogenous mtDNA levels (rho+) in the same 143B background (S5B Fig).

The increased methylation of the DNA is consistent with the increased levels of SAM and with the kinetics of availability of SAM/SAH amounts over time. However, changes in other TCA metabolites could also play a role in this phenotype. For example, α-ketoglutarate is a cofactor of the Ten-eleven translocation (TET) enzymes, which are involved in the DNA demethylation reactions. Also, succinate, fumarate, and 2-hydroglutarate (2-HG) can compete with α-ketoglutarate in the active site of the TETs, inhibiting their function [25]. Thus, decreased α-ketoglutarate, increased succinate, fumarate, and/or 2-HG could also lead to hypermethylation of the DNA. However, no changes in the levels of α-ketoglutarate were observed (S3 Data), and no increases in the succinate or fumarate to α-ketoglutarate ratios were identified over the time course of the experiments (Fig 2D). Despite the fact that 2-HG increased as mtDNA was depleted, only a small change was observed at day 6, and maximal accumulation was observed at day 9 (Fig 2E), which is inconsistent with the kinetics of DNA hypermethylation (Fig 2C). Levels of methylated cytosines (5meC) were increased, while no changes in the levels of 5-hydroxy-methyl-cytosine (5hmeC)—the product of TET reaction—were identified in cells chronically depleted of mtDNA (S5C and S5D Fig). We also showed enhanced DNA methyltransferase (DNMT) activity (Fig 2F). Collectively, these data are in support of DNA hypermethylation resulting from increased DNA methylation and not from inhibition of the demethylases.

### Changes in DNA methylation occur prevalently in DEGs

In order to determine whether the changes in global methylation influenced gene expression, we cross-referenced the coordinates of the promoters differentially methylated at days 3, 6, or 9 with those of the DEGs. We found that 1,627 (approximately 57%) of the DEGs showed significant alterations in their promoter methylation when compared to day 0 (S4 Data). The number of differentially methylated DEGs increased over time from 63 at day 3 (27%), 978 (46%) at day 6, and 879 (70%) at day 9 (S5E Fig). The odds ratio (OR) of a gene being differentially expressed and having a change in its promoter methylation was OR = 0.81, *p* < 0.01 (S5F Fig), suggesting that incidence of promoter DNA methylation changes is different for DEGs and genes not differentially expressed.

To better understand the relationship between differential methylation, gene expression, and mitochondrial dysfunction, we performed IPA on the DEGs that were differentially methylated. This analysis revealed that genes involved in key pathways that responded to mtDNA depletion were targets of differential methylation. For instance, at day 3, the 63 differentially methylated and expressed genes enriched for methionine degradation; at day 6, for cholesterol biosynthesis; and at day 9, for the metabolism of several amino acids, cholesterol, and the TCA (S5 Data). Similar findings were observed when evaluating the 143B rho0 cells chronically depleted of mtDNA that also showed hypermethylation of the DNA. In those cells, 621 DEGs were also differentially methylated (S6 Data) and enriched for pathways involved, for instance, in folate transformations (S5G Fig). While DNA methylation is not the only parameter governing gene expression, we attempted to define the level of concordance between the changes in DNA methylation status over time with the directionality of expression of the DEGs harboring those changes. Whether we combined the entire methylation profile of genes or considered only promoter marks, the concordance ranged from 30%–50% over the 9 days of mtDNA depletion (S5H Fig). Taken together, these findings suggest a correlation between DNA methylation changes and the expression of a fraction of DEGs responding to progressive mitochondrial dysfunction.

### NADH oxidation in the mitochondria links polyamine and methionine metabolism to the TCA cycle and DNA methylation

It is possible that the mechanism connecting mtDNA depletion to SAM and DNA hypermethylation involves serine biosynthesis and 1C-folate remodeling, which in turn can affect the methionine cycle [8,9]. While this is feasible, the fact that choline/betaine are engaged in maintaining methionine salvage independent of folate would argue against this possibility. Alternatively, the methionine cycle may be directly affected by mtDNA depletion through changes in both methionine and polyamine metabolism. These molecules are not only linked in the regulation of SAM levels [10], but they can provide intermediates such as pyruvate, succinyl-CoA (a precursor of succinate), and succinate to the TCA in their catabolic pathways. An increase in their degradation to feed the TCA could set a cascade of compensatory changes that impacts the SAM pool. To test this hypothesis, we took advantage of the DN-POLG cells overexpressing NDI1/AOX, which are cells that have the ability to oxidize NADH and maintain TCA flux despite the complete loss of mtDNA [4]. We reasoned that if the methionine cycle is directly impacted by the TCA, in these cells methionine-associated intermediates should not be changed.

**Fig 3:**
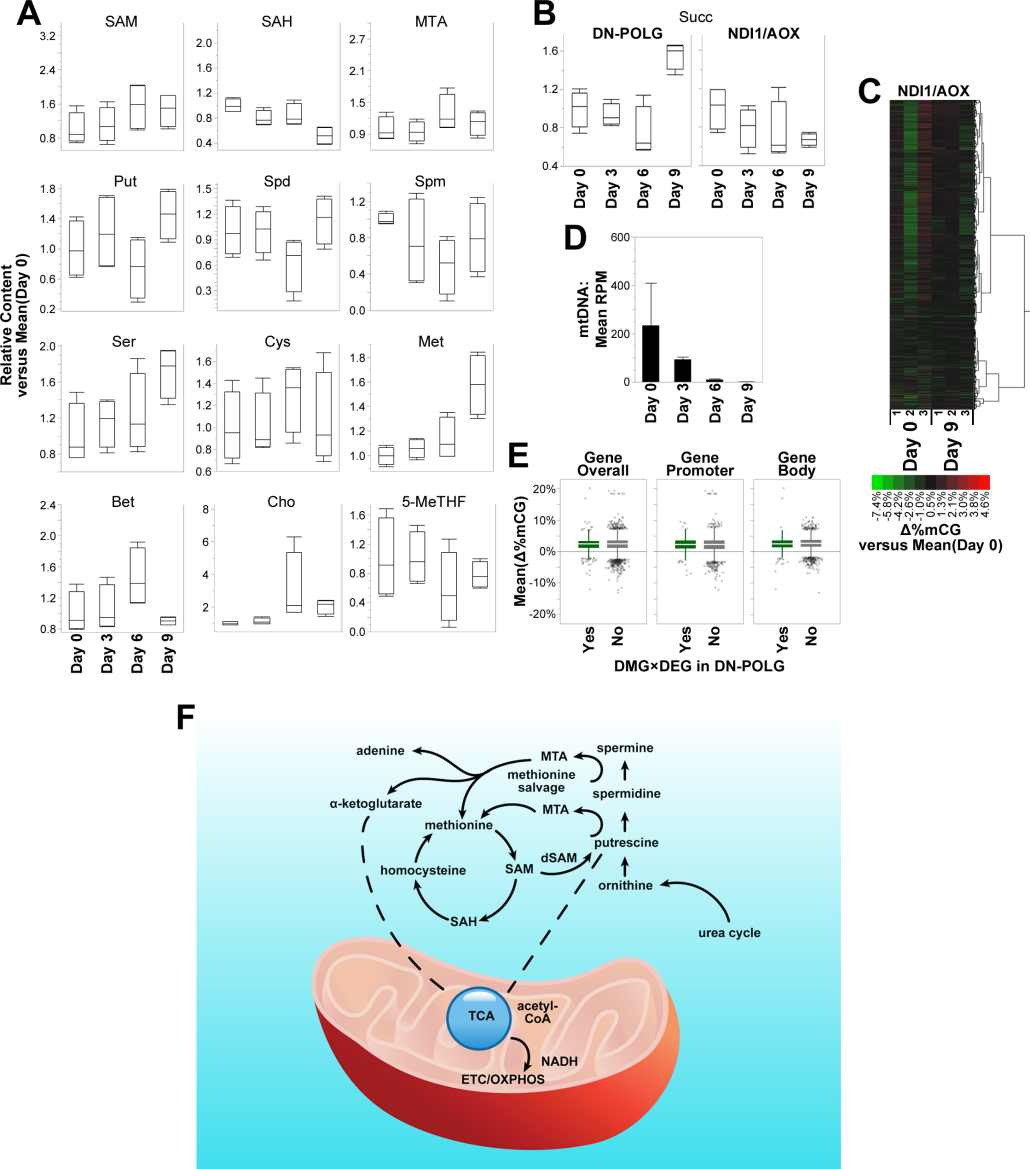
NDI1/AOX ectopic expression maintains DNA methylation while not completely rescuing metabolism. Metabolomics was performed in NDI1/AOX cells at days 0, 3, 6 and 9; *N* = 4 per time point. Differentially enriched metabolites were identified based on log2-transformed fold-changes in arbitrary detection units versus the mean at day 0 (y-axis) in each time point during doxycycline treatment (x-axis) by a two-way ANOVA test. (A) Box plots of relative content versus day 0 (IQR-outlier format) depict distribution of individual replicates in NDI1/AOX cells within statistical groups for SAM, SAH, MTA, Put, Spd, Spm, Ser, Cys, Met, Bet, Cho, and 5-MeTHF. (B) Box plots of relative content versus day 0 (IQR-outlier format) depict distribution of individual replicates within statistical groups for Succ in DN-POLG cells (left) and their NDI1/AOX counterparts (right). (C) Δ%mCG in NDI1/AOX cells at days 0 and 9 for the same probes identified as DML in DN-POLG cells by HM-450K BeadArrays [see Fig 2C]; hypermethylation (red) or hypomethylation (green). *N* = 3 per time point. (D) Average normalized read counts (bar plot: mean ± SEM) of mtDNA fragments obtained by next-generation sequencing of whole-cell DNA for NDI1/AOX cells; *N* = 2 per timepoint. (E) Box plots (IQR-outlier format) of average DNA methylation differences between day 0 and day 9 at independently identified DML in NDI1/AOX cells overlapping the genomic range (overall), only promoters, or only bodies of DN-POLG DEGs; separate box plots depict measurements from DN-POLG DEGs that are DMG or not in DN-POLG cells also. Underlying data are reported in S3 Data for (A) and (B); S4 Data for (C) and (E); and S1 Data for (D). (F) Model for the cross talk between methionine salvage, polyamine synthesis and the TCA cycle with DNA methylation: loss of mtDNA decreases TCA flux, which in turn sets a cascade of transcriptional and metabolic changes centered largely on amino acid degradation to maintain TCA cycle output. Degradation of methionine and Put, both of which can feed into the TCA cycle, are among the first changes detected. Put levels are regulated by ornithine, which is provided by the recycling of NH_3_ resulting from amino acid degradation. Put is also a precursor of Spd and Spm, both of which require dSAM for their synthesis. The main byproduct is MTA, which needs to be quickly recycled, given its accumulation is toxic; the salvage of MTA recycles methionine, a cycle that is also maintained by folate-independent Cho/Bet when mtDNA is depleted. Recycling of MTA also generates adenine, which can enter the purine pool, and α-ketoglutarate that can feed the TCA. By maintaining NADH oxidation in the mitochondria, flux through the TCA cycle is largely normalized, “turning off” the polyamine/MTA salvage response that in turn decreases levels of SAM. Decrease in degradation of amino acids to feed the TCA diminishes flux through the urea cycle, decreasing the input of ornithine to Put biosynthesis. 5-MeTHF, 5-methyltetrahydrofolate; acetyl-CoA, acetyl coenzyme A; AOX, alternative oxidase; Bet, betaine; Cho, choline; Cys, cysteine; Δ%mCG, DNA methylation differences versus average of day 0; DEG, differentially expressed gene; DMG, differentially methylated gene; DML, differentially methylated loci; DN-POLG, dominant-negative DNA polymerase gamma transgene; dSAM, decarboxylated S-adenosylmethionine; ETC; electron transport chain; Met, methionine; MTA, 5-methylthioadenosine; NADH, nicotinamide adenine dinucleotide reduced; NDI1, nicotinamide adenine dinucleotide reduced dehydrogenase-like 1; OXPHOS, oxidative phosphorylation; Put, putrescine; RPM, reads per million reads; SAH, S-adenosylhomocysteine; SAM, S-adenosylmethionine; Ser, serine; Spd, spermidine; Spm, spermine; Succ, succinate; TCA, tricarboxylic acid.

We reanalyzed the metabolomics data that we previously generated with the NDI1/AOX cells [4] using the same criteria as for the DN-POLG cells (S3 Data). We then focused on the intermediates associated with the methionine, serine, folate, and polyamine pathways. We found that in the NDI1/AOX cells, the levels of SAM, SAH, MTA, and the polyamines were maintained over time (Fig 3A); the levels of serine, cysteine, methionine, betaine, choline, and folate followed the same pattern as was observed with the DN-POLG cells (compare Figs 3A and 1E–1G). Most notably, levels of succinate, which were increased at day 9 in the DN-POLG, were decreased in the NDI1/AOX cells (Fig 3B). Taken together, these results support the hypothesis that polyamine and methionine metabolism are directly responding to changes in TCA flux, likely as contributors of succinate. Furthermore, these data suggest that serine biosynthesis and folate-1C remodeling caused by mtDNA depletion are not responding to changes in NADH oxidation or TCA flux.

We also evaluated whole genome methylation using the Illumina 450K platform in NDI1/AOX cells. We used cells at days 0 and 9, since we gauged that mtDNA would be fully depleted at this latter time and would provide the largest effect. Remarkably, no significant changes in DNA methylation were observed in the cells expressing NDI1/AOX, despite complete loss of mtDNA (Fig 3C and 3D). We then focused specifically on the coordinates of the 1,626 DEGs that were differentially methylated in the DN-POLG cells at day 9 (S5A Fig). However, we found that average DNA methylation change in those sites was only approximately 2% in the NDI1/AOX cells (Fig 3E). Hence, we conclude that changes in polyamine synthesis and the MTA salvage pathway, which in turn affect SAM levels, seem to be critical for differential DNA methylation in the nucleus of DN-POLG cells.

### Lack of changes in DNA methylation are associated with the prevention of differential gene expression, even in the context of complete mtDNA loss

We performed gene expression analysis in the NDI1/AOX cells using microarrays in order to determine whether the promoter methylation status has the potential to impact the differential expression of the 879 genes identified in the DN-POLG cells at day 9. Unexpectedly, we found no significant DEGs in the NDI1/AOX cells between days 0 and 9 when adjusting for false discovery rate (FDR; S7 Data). Relaxing statistical thresholds based on pairwise comparisons without multiple testing corrections revealed 23 genes that were differentially expressed between days 0 and 9 (S6 Data), 4 of which were also differentially expressed in the DN-POLG at day 9, as gauged by RNA-seq. To rule out that these results were due to a lack of sensitivity of microarrays to detect the relatively small changes in gene expression identified by RNA-seq, we performed microarrays in DN-POLG cells at days 0 and 9. We found 1,408 genes with adjusted *p* ≤ 0.05 that were differentially expressed between days 0 and 9 in this cellular background (S7 Data). These results indicate that it is the maintenance of NADH oxidation in the mitochondria, in the context of mtDNA depletion, that prevents the differential expression of genes.

To better understand the effects of NDI1/AOX expression in the presence of mtDNA, we next compared the microarray data from DN-POLG cells with those from NDI1/AOX cells at day 0. Again, no DEGs were detected when adjusting for FDR. Using unadjusted *p*-values, we found 842 genes that were differently expressed between the 2 cell types at day 0 (S8 Data). However, Kyoto Encyclopedia of Genes and Genomes (KEGG) pathway analysis identified little to no overlap to the findings obtained when utilizing the DEGs identified in the DN-POLG when mtDNA was depleted (S8 Data). Thus, we conclude that expression of NDI1/AOX does not cause significant off-target effects. Nevertheless, the maintenance of NADH oxidation provided by these enzymes is sufficient to prevent the DNA methylation and transcriptomic changes that result from mtDNA depletion, independent of mitochondrial ATP production or the ΔΨm, which were not rescued by NDI1/AOX expression [4].

## Discussion

Our understanding of how changes in mitochondrial function can impact the epigenetic control of gene expression in the nucleus is still incomplete. Despite the fact that mitochondrial dysfunction has been shown to affect histone modifications and DNA methylation, mechanistic links between these processes have not been elucidated, particularly in terms of methylation reactions. Earlier studies proposed a prominent role for ROS or 2-HG as inhibitors of the DNA or histone demethylases [26–29]. However, a direct demonstration that the demethylases are inhibited as mitochondria become dysfunctional is still lacking. In this study, we used a novel cell culture system of progressive mtDNA depletion, an isogenic counterpart cell line that was engineered to maintain NADH oxidation, despite loss of mtDNA, and a widely used cell line that is chronically depleted of mtDNA to demonstrate that (i) the methionine cycle responds to loss of TCA function; (ii) salvage pathways of methionine, including through MTA, are engaged in the context of mtDNA loss; (iii) DNA hypermethylation is associated with higher SAM concentrations, 5meC levels, and DNMT activity; (iv) changes in DNA methylation occur predominantly in genes differentially expressed as a result of mtDNA depletion; and (v) genes involved in key pathways responding to mtDNA depletion are targets of differential methylation. Collectively, these findings provide new mechanistic insights that connect mitochondrial function and epigenetics in a way that is likely to have broader relevance to health and disease. In view of their potential impact, further studies to validate and explore these findings in different cell types and in vivo are warranted.

Our transcriptomic analysis revealed a dynamic relationship between the loss of mtDNA and the genes that respond to the resulting mitochondrial dysfunction. Our results confirm the serine response that was recently reported by others [8,9,13], but we found that it involves a series of additional amino acids, including methionine. We also identified that inhibition of fat metabolism, perhaps as an additional means of sparing acetyl-CoA, started early in the progression of mitochondrial dysfunction. The identification of methionine degradation as an early response to mtDNA depletion was unexpected. To our knowledge, no direct link between these processes have been previously reported. Our data with the NDI1/AOX cells clearly suggest that resuming TCA flux can turn off the methionine response involving the salvage pathway through MTA. Methionine salvage from MTA can also contribute to the generation of α-ketoglutarate, and because it is intimately linked to polyamine synthesis, where putrescine can generate succinate, we propose a model in which the methionine cycle responds to mtDNA depletion based on changes in TCA intermediates (Fig 3F). At the same time, MTA can also contribute to the purine pool, whose imbalance seems to be an early response to mtDNA loss, perhaps as an additional means to maintain homeostasis.

It had been previously shown that, at least in cancer cells, serine contributes to SAM, DNA, and RNA methylation by de novo ATP synthesis [30]. However, maintenance of TCA flux in the NDI1/AOX cells had no impact on serine biosynthesis under our experimental conditions, which rules out that serine is limiting for SAM levels under conditions of mtDNA depletion. It is currently unclear how (and whether it is that) changes in flux through the TCA cycle or changes in the levels of particular TCA metabolites are sensed outside of the mitochondria. Given that acetyl-CoA is an important intermediate of the TCA cycle, which is used both for biosynthetic purposes and for posttranslational protein modifications [31,32], it is possible that its levels are sensed by other parts of the cell. Changes in these levels initiate an entire cascade to ultimately preserve a level of acetyl-CoA that is compatible with maintenance of key cellular functions. We suggest that a sensitive but yet-unidentified pathway that senses and regulates TCA intermediates, such as acetyl-CoA (or flux), within the mitochondria must exist and efficiently communicate to the rest of the cell.

The activation of serine biosynthesis and the remodeling of mitochondrial folate and 1C metabolism toward transsulfuration to produce cysteine and glutathione have recently been the subject of various studies. It was shown that this pathway is consistently engaged upon in vitro and in vivo mtDNA depletion, by the inhibition of the ETC with agents such as rotenone or antimycin C, or by compounds that alter protein homeostasis [8,9,13,33]. Our data revealed that the remodeling of 1C metabolism toward transsulfuration is a response to overt mitochondrial dysfunction but does not take place when the organelle is still able to maintain ETC function. It remains unclear why the mitochondrial folate pool and serine biosynthesis are engaged upon mitochondrial dysfunction, although it has recently been shown that mammalian target of rapamycin (mTOR) and the transcription factor ATF4 are involved in these processes [8,13,33]. While we envision that alterations in 1C metabolism can effectively alter nucleotide pools, redox, and methylation reactions—thus potentially impacting cell cycle, transcription, replication, and signaling concomitantly—the exact signal that arises from mitochondria remains to be identified. It has been proposed that ATF4-dependent serine biosynthesis arises from oxidative stress due to a stalled respiratory chain [8]. However, our data do not support this hypothesis, since we found no changes in respiratory function at day 3 [4] and, most importantly, that maintenance of NADH oxidation in the NDI1/AOX cells does not turn off the serine biosynthesis response. Further studies will be required to resolve this issue.

Our finding that there is an extensive cellular metabolic rewiring associated with mitochondrial dysfunction centered on amino acid and lipid metabolism was not fully surprising. What was unexpected was that the maintenance of NADH oxidation in NDI1/AOX cells prevented, most significantly, the changes in methionine metabolism and polyamine synthesis. To our knowledge, this is the first report that directly connects these pathways to mitochondrial function. The roles of polyamines in cell biology are still poorly understood and are linked to effects on cell proliferation, chromatin configuration, gene transcription, and even mitochondrial calcium homeostasis [34,35]. Whether the changes in polyamine levels we identified to be associated with mitochondrial dysfunction are sufficient to affect any of these processes requires further studies. It is worth noting that a mouse completely deficient in spermine synthase activity was identified in a cohort of female irradiated offspring [36,37]. The complete loss of polyamines is embryonic lethal in vivo [38]. Most interestingly, these mice have many phenotypes that resemble mitochondrial diseases, including deafness, sterility, neurological abnormalities, and reduced life span [39,40], which supports a potential link between polyamine synthesis and mitochondrial health.

Finally, our data revealed that many of the genes that are differentially expressed in response to mtDNA depletion are also differentially methylated. Moreover, we showed these changes in methylation and gene expression can be prevented by maintaining NADH oxidation in the NDI1/AOX cells. These findings support the notion that the epigenetic changes caused by mtDNA depletion are intimately associated with the differential expression of genes in the DN-POLG cells. It is possible that posttranscriptional modifications of specific proteins, and not transcriptional changes of specific genes, account for the lack of differential gene expression in the cells overexpressing the NDI1/AOX transgenes. However, we think this is unlikely to be the case. Alternatively, it can be argued that it is the metabolic rescue provided by NADH oxidation in the mitochondria that is directly responsible for turning off the transcriptional response in these cells. Given the continued activation of the metabolism of serine, folates, and others, despite maintenance of TCA flux, we do not favor this possibility. More studies are required to better address these issues.

The remarkable parallels between our in vitro results and the data obtained with in vivo mouse models and patient samples of mitochondrial disorders [9,33] suggest that our findings may be relevant to human health. In fact, careful analysis of the metabolic data from heart and muscle of the Deletor mouse strain indicate that the methionine salvage pathway and polyamine synthesis are altered based on increased steady state levels of choline, betaine, ornithine, and MTA (S6 Fig) [9]. Similar findings were observed in the liver of another mouse model of mtDNA abnormalities driven by a thymidine kinase mutation [41] in which MTA and adenosine levels are higher than wild-type controls (S6 Fig). However, whether the DNA is also hypermethylated in those models and which genes may be affected by it remain to be addressed. It also remains to be determined the extent to which the flux from polyamines to the methionine cycle end up in methylation reactions and whether these changes are drivers or contributors of the overall response to mtDNA depletion. It is also unclear whether these same effects occur in the context of other types of mitochondrial dysfunction. This will be especially important for studies to reveal how environmental toxicants that target the mitochondria change the biology of the cell. Irrespective of these limitations, our findings have the potential to fundamentally change our understanding about the role and impact of mitochondrial metabolism in health and disease.

## Materials and methods

### Cells

HEK293T cells carrying a tetracycline (Tet)-on inducible DN-POLG from [42] were used to generate derivatives also ectopically expressing AOX and NDI1 and cultured as described previously [4]. The osteosarcoma cell line 143B and its rho0 derivative, graciously obtained from Dr. Eric Schon at Columbia University, were routinely grown in DMEM high glucose (4.5 g/L) supplemented with 10 mM pyruvate, 50 μg/mL of uridine, 10% FBS, and 1% penicillin/streptomycin under 37°C and 5% CO_2_. Except for ChIP-seq experiments (*N* = 2) all experiments were performed on *N* = 3 independent biological replicates.

### RNA extraction

RNA was extracted from 3 independent cell cultures of DN-POLG cells at days 0, 3, 6, and 9 using RNAeasy Mini and QIAshredder kits (QIAGEN) and was used for both RNA-seq and microarrays; RNA from NDI1/AOX cells was obtained at days 0 and 9 and used for microarrays only. In all cases, samples from 3 independent experiments were collected (*N* = 3 per time point per cell model).

### Quantification of DNMT activity in 143B cells

DNMT activity was assayed using radioactive filter-binding assay, as previously described [43]. Briefly, 143B cells from rho+ or rho0 cells were lysed and the nuclear fraction enriched using differential centrifugation. Then, cell lysates were used to monitor the incorporation of tritiated (3H) methyl groups into a poly-IC duplex DNA oligonucleotide. The unreacted (methyl-3H) was separated from the radiolabeled DNA using filter binding. The 3H–CH3-containing duplex DNA was then quantified by liquid scintillation. Data were normalized to protein content and presented relative to the detected activity in rho+ cells. Samples from 3 independent replicates were collected (*N* = 3 per 143B derivative).

### Gene expression experiments by RNAseq in DN-POLG cells

RNA from DN-POLG cells at days 0, 3, 6, and 9 (*N* = 3 each timepoint) was poly-A-selected and sequenced with a HiSeq 2000 system (Illumina). Following 3′ adapter trimming and base-calling filtering (phred score > 20), we obtained approximately 100 million 126-nt paired-end reads per individual sample (7–8 flow cell lanes/sample), which were aligned to the hg19 human reference genome (Genome Reference Consortium GRCh37 from February 2009) [44] with TopHat-Fusion function [45]. Composite RPKM counts within genomic coordinates of 20,304 nonhaplotype HGNC-annotated genes were used to calculate gene expression differences based on log2-transformed fold-change (log2FC) relative to average gene RPKM at day 0 at a significance level *p* < 0.05 adjusted for multiple comparisons [46].

### Detection of DEGs by RNA-seq

DEGs were detected using weighed two-way ANOVA (gene × time) of log2-transformed expression fold change measurements with respect to the average composite RPKM at day 0 (log2FC); *N* = 12 (3 biological replicates per time point). Gene-wise log2FC values were weighed by a relative metric of sequencing representation (cumulative hazard of significance scores from gene-wise RPKM rate modeling with an exponential distribution and inverse link function). A total 2,854 HGNC-annotated DEGs were detected in DN-POLG cells at a significance level *p* < 0.05 adjusted for multiple comparisons [46], filtering against a minimum gene-wise effect size δ_log2FC_ > 0.3 × σ_log2FC_, and post hoc pairwise significance (Student *t* test *p* < 0.05) between log2FC values at days 3, 6, or 9 versus day 0. For gene-level effect size filtering, δ_log2FC_ = 0.3 × σ_SSR_ is 5% of the 6σ-spread log2FC regression error with respect to a gene’s grand mean (where [σ_SSR_]^2^ = [SSR_log2FC_] / [N– 1]) compared to 5% of the 6σ-spread in measurement error about the mean log2FC at each time point in the gene (where [σ_log2FC_]^2^ = [SSE_log2FC_] / [N-1]).

### Gene expression experiments by microarray technology and data analyses

For microarrays analysis of gene expression, the Affymetrix Human Genome U133 Plus 2.0 GeneChip arrays were used. Samples were prepared as per manufacturer’s instructions. Arrays were scanned in an Affymetrix Scanner 3000 and data were obtained using the GeneChip Command Console and Expression Console Software (AGCC, Version 3.2; Expression Console, Version 1.2) using the MAS5 algorithm to generate CHP-extension files. ANOVA was used to identify statistical differences between means of groups at α < 0.05 level among HG-U133 Plus 2.0 probe sets unambiguously mapped to UCSC known gene transcripts.

### Detection of differentially enriched metabolites

Differentially enriched metabolites were detected for DN-POLG and NDI1/AOX cells separately using two-way ANOVA (metabolite × time) of log2-transformed relative changes in abundance versus untreated cells (log2RC); *N* = 16 (4 biological replicates per time point in each DN-POLG and NDI1/AOX); refer to previously published original data elsewhere [4]. Data with significance level *p* < 0.05 adjusted for multiple comparisons [46] were then filtered against minimum effect size δ_log2RC_ ≥ 0.3 × σ_log2RC_ = 0.20 (approximately 1.15-fold change). The δ_log2RC_ corresponds to the smallest estimate between DN-POLG and NDI1/AOX cells of metabolome-wide effects at 5% of the 6σ-spread in log2RC measurement error across all metabolite × time groups (where [σ_log2RC_]^2^ = [SSE_log2RC_] / [*N*– 1]).

### DNA methylation arrays and data analyses

Genomic DNA was extracted from 3 independent cell cultures of DN-POLG cells at days 0, 3, 6, and 9 and NDI1/AOX cells at days 0 and 9 and bisulfite-converted using an EZ DNA Methylation kit (Zymo Research) following the manufacturer’s protocol. Differential methylation at the CpG dinucleotide level was conducted using Human Methylation450 v1 BeadChip arrays (Illumina) following the InfiniumHD methylation protocol. Data were obtained using Illumina’s GenomeStudio software (version 2011.1) using no background subtraction and no normalization parameters. Probes in a probe × cell × time block with >1 failed reads or >1 outliers (1.5 IQR rule on residuals around probe × cell × time sample means) were discarded from analysis. Probe-level groups with *N* = 2 after failed read or outlier filtering were brought to *N* = 3 by substitution with probe × cell × time trimmed mean values adjusted by imputed array-wise residual estimates using the pairwise correlation matrix of cell × time statistical groups. Probe methylation percentage was quantified from fluorometric signal intensities of methylation (mCG) and unmethylation (CG) in terms of ß = [mCG/(mCG+CG)] × 100. ANOVA was used to identify statistical differences between the means of groups at a significance level *p* < 0.05 adjusted for multiple comparisons [46] using JMP software (Version 11) followed by post hoc pairwise significance testing (*p* < 0.05) with respect to day 0 in DN-POLG or NDI1/AOX cells.

## Supporting information

S1 Fig Experimental design and measures of reproducibility in DN-POLG and NDI1/AOX model of dox-inducible mtDNA depletion. (A) Schematic representation of experimental procedure and data integration. Culture splits were performed every 3 days, corresponding to observed interval needed for noninduced DN-POLG or NDI1/AOX starter cultures to return to confluence after 1:2 subcultivation. As depicted, each cell culture round going from one single starter culture to individual cultures at day 0 (1 flask), 3 (1 flask), 6 (2 flasks, pooled), or 9 (4 flasks, pooled) of continuous supplementation with doxycycline (10 ng/mL) represents a single biological replicate. Independent biological replicates per cell model and per timepoint were produced for different assays: transcriptomics, *N* = 3; metabolomics, *N* = 4; DNA methylation, *N* = 3. (B–D) Reproducibility of biochemical output among DN-POLG biological replicates as shown by pairwise concordance and Pearson’s correlation *r* within different assays: (B) RNA-seq (Log_10_[RPKM], poly[A]-enriched RNA, uniquely aligned reads to hg19 reference genome; days 0, 3, 6, and 9); (C) Illumina HM-450K DNA Methylation BeadArrays (% mCG/CG, bisulfite-converted DNA; days 0, 3, 6, and 9); and (D) Metabolon mass spectrometry (Log_10_[Content], arbitrary units; days 0, 3, 6, and 9). (E–G) Reproducibility of biochemical output among NDI1/AOX biological replicates, as shown by pairwise concordance and Pearson’s correlation *r* within different assays: (E) Affymetrix HG-U133 Plus 2.0 Microarrays (Log_10_[Intensity], Total RNA; days 0 and 9); (F) Illumina HM-450K DNA Methylation BeadArrays (%mCG/CG, bisulfite-converted DNA; days 0 and 9); and (G) Metabolon mass spectrometry (Log_10_[Content], arbitrary units; days 0, 3, 6, and 9). Underlying data are reported in S1 Data for (B); S4 Data for (C) and (F); S3 Data for (D) and (G); and S7 Data for (E). %mCG, percentage of DNA methylation; AOX, alternative oxidase; DN-POLG, dominant-negative DNA polymerase gamma transgene; mtDNA, mitochondrial DNA; NDI1, nicotinamide adenine dinucleotide reduced dehydrogenase-like 1; RNA-seq, RNA sequencing; RPKM, reads per kilobase per million.
(TIF)

S2 Fig Validation of RNA-seq measurements in DN-POLG cells by quantitative PCR. (A) Left upper panel depicts concordance of relative expression estimates normalized to housekeeping ACTB between RNA-seq and qPCR experiments for 12 nuclear-encoded DEGs and 3 mtDNA-encoded genes; increasingly darker shades of brown (nuclear-encoded) and gray (mtDNA-encoded) depict relative gene expression values at days 3, 6, and 9 each versus day 0. qPCR was performed from cDNA templates derived using the same total RNA extracts for both techniques (*N* = 3 per timepoint) and were performed in technical triplicates for each of the 3 biological replicates. Right top panels show graphs for mtDNA-encoded genes while the bottom panels depict data from nuclear DNA-encoded transcripts. Underlying data are reported in S1 Data. (B) Top 5 significantly enriched upstream regulators for upregulated and downregulated DEGs in DN-POLG cells at days 3, 6, and 9 of dox-inducible mtDNA depletion, per Ingenuity Pathway Analysis. ACTB, ß-actin; DEGs, differentially expressed genes; DN-POLG, dominant-negative DNA polymerase gamma transgene; mtDNA, mitochondrial DNA; qPCR, quantitative polymerase chain reaction; RNA-seq, RNA sequencing.
(TIF)

S3 Fig Differentially enriched metabolites and significantly associated metabolic pathways in DN-POLG cells. Metabolomics was performed in DN-POLG cells at days 0, 3, 6, and 9; *N* = 4 per time point. Differentially enriched metabolites were identified based on log2-transformed fold-changes in arbitrary detection units versus the mean at day 0 by a two-way ANOVA test (metabolite × time) at an adjusted Benjamini-Hochberg *p* ≤ 0.05, and are detailed in S3 Data. (A) Number of differential metabolites identified in DN-POLG cells at days 3, 6, and 9 of doxycycline supplementation compared to day 0 (circle plots, top), and their overlap between time points (Venn diagram, bottom). (B) Heatmap of significantly represented canonical metabolic pathways per Ingenuity Pathway Analysis [−log(p) > 1.3] based on differentially enriched metabolites in DN-POLG cells at days 3, 6, and 9 of dox-inducible mtDNA depletion. Coloring of listed pathway names correspond to clades of pathways inferred by unsupervised clustering of enrichment scores across timepoints. (C–G) Data integration of average log_2_-fold changes relative to day 0 in gene expression (down: green, up: red; numerical values reported in S1 Data) and metabolite enrichment (down: blue, up: red; numerical values reported in S3 Data) with PathVisio engine for (C) nucleotide metabolism, (D) Methionine De Novo and Salvage Pathway, (E) One-Carbon Metabolism and Related Pathways, (F) Amino Acid Interconversion, and (G) Amino Acid Metabolism. (H) Depiction of three metabolic nodes identified as the main drivers of the response to mtDNA depletion in DN-POLG cells by day 3, per Ingenuity Pathway Analysis. acetyl-Coa, acetyl coenzyme A; DN-POLG, dominant-negative DNA polymerase gamma transgene; mtDNA, mitochondrial DNA; TCA, tricarboxylic acid.
(PDF)

S4 Fig Doxycycline supplementation induces loss of mtDNA copy number and expression in DN-POLG cells over 9 days. (A) Average normalized read counts (RPM; bar plots: mean ± SEM) of mtDNA fragments obtained by next-generation sequencing of whole-cell DNA for DN-POLG cells; *N* = 2 per timepoint. (B) Average normalized read counts (reads per kilobase per million reads, RPKM; bar plots: mean ± SEM) of mtDNA transcripts (mtRNA) obtained by RNA-seq for DN-POLG cells; *N* = 3 per timepoint. Underlying data are reported in S1 Data. DN-POLG, dominant-negative DNA polymerase gamma transgene; mtDNA, mitochondrial DNA; RNA-seq, RNA sequencing; RPKM, reads per kilobase per million reads; RPM, reads per million reads.
(TIF)

S5 Fig DNA methylation levels in DN-POLG cells are changed in the course of mtDNA depletion. (A) Violin plots depict Δ%mCG for DML observed in DN-POLG cells by HM-450K BeadArrays broken down by probe annotated location: IGR, TSS 1500 and TSS 200, 5'UTR, 1st exon (exon 1 as per annotated reference genome), body (gene body), and 3'UTR. Number of DML at each time point is shown at the top of each plot frame within each time point and probe location class. (B) Unsupervised hierarchical clustering showing the percentage of methylation of individual locus, presented in each row, in either rho+ or rho0 cells based on Illumina 450K methylation arrays; *N* = 3. (C) Immunofluorescence using antibodies again 5meC or 5hmeC in both cell types; images taken with confocal microscope; blue—DAPI-stained nucleus; controls containing only primary or only secondary antibodies or background fluorescence are omitted. (D) Dot blots probing levels of 5meC or 5hmeC were performed in technical triplicates; *N* = 12. (E) Number of DMEGs in DN-POLG cells at each time point is shown above each panel. All identified differentially methylated probes relative to day 0 associated with DEGs in DN-POLG cells are shown. X-axis depicts the degree of methylation change while the y-axis shows the expression change for each respective DEG. (F) Expected versus observed number of genes that bear or do not bear changes in DNA methylation relative to DEGs in DN-POLG cells; *p*-value was calculated using chi-squared test. (G) Bar graph shows metabolic pathways identified enriched in 143B rho0 cells chronically depleted of mtDNA based on 621 DEGs that were also differentially methylated, per Ingenuity Pathway Analysis; the top x-axis depicts log_10_-tranformed significance score of enriched pathways, whereas the bottom ratio x-axis (bottom) and line plot (orange) reflects the proportion of members within each pathway that are present in the dataset. (H) Concordance between gene expression directionality (y-axis) and change in DNA methylation levels (x-axis) are presented for each time point. The number of DEGs in each quadrant is also indicated; colors depict quantile density for “gene overall” plots. Values of Δ%mCG for “gene overall” plots equal the average of all probes inside genes, probes in their 5'–or 3' untranslated regions, and promoters; Δ%mCG values are also split into averages of promoter only [P, blue] or within-gene-body [B, red] probes. Underlying data are reported in S4 Data for (A), (E), (F), and (H); in S6 Data for (B); and in S1 Data for (E), (F), and (H). 5hmeC, 5-hydroxy-methyl-cytosine; 5meC, 5-methyl-cytosine; 5'UTR, 5- untranslated region; Δ%mCG, DNA methylation differences versus average of day 0; DEGs, differentially expressed genes; DMEGs, differentially methylated and expressed genes; DML, differentially methylated loci; DN-POLG, dominant-negative DNA polymerase gamma transgene; IGR, intergenic region; mtDNA, mitochondrial DNA; TSS 1500, transcription start site 1,500
(PDF)

S6 Fig Metabolite increased in the DN-POLG cells are also changed in mice models of mtDNA depletion. Graphs depict levels of metabolites in Deletor mice (skeletal muscle or heart) based on the fold-change relative to wild-type littermates, as reported in [9]. TK2 data were calculated based on normalized metabolite values from the KOs versus wild-type counterparts, as reported in [41]. DN-POLG, dominant-negative DNA polymerase gamma transgene; KO, knockout; MTA, 5-methyl-thioadenosine; mtDNA, mitochondrial DNA; TK2, timidine kinase 2
(TIF)

S1 Data Differentially expressed genes detected in DN-POLG cells by RNA-seq during 9-day time course of dox-inducible mtDNA depletion. ACTB, ß-actin; DN-POLG, dominant-negative DNA polymerase gamma transgene; mtDNA, mitochondrial DNA; RNA-seq, RNA sequencing; RPKM, reads per kilobase per million; ChrM, chromosome M (mitochondrial).
(XLSX)

S2 Data Significantly enriched pathways in DN-POLG at days 3, 6, and 9 of dox-inducible mtDNA depletion for upregulated and downregulated DEGs via Ingenuity Pathway Analysis. DEGs, differentially expressed genes; DN-POLG, dominant-negative DNA polymerase gamma transgene; mtDNA, mitochondrial DNA.
(XLSX)

S3 Data Differentially enriched metabolites (log_2_-ratio versus day 0) in DN-POLG and NDI1/AOX cells by metabolomics assays (Metabolon) during 9-day time course of dox-inducible mtDNA depletion. AOX, alternative oxidase; DN-POLG, dominant-negative DNA polymerase gamma transgene; mtDNA, mitochondrial DNA; NDI1, nicotinamide adenine dinucleotide reduced dehydrogenase-like 1
(XLSX)

S4 Data DEGs with differential DNA methylation in DN-POLG cells per time point during mtDNA depletion. AOX, alternative oxidase; Δ%mCG, DNA methylation differences versus average of day 0; DEGs, differentially expressed genes; DN-POLG, dominant-negative DNA polymerase gamma transgene; mtDNA, mitochondrial DNA; NDI1, nicotinamide adenine dinucleotide reduced dehydrogenase-like 1
(XLSX)

S5 Data Significantly enriched pathways at days 3, 6, and 9 of dox-inducible mtDNA depletion for DEGs with differential DNA methylation in DN-POLG cells via IPA. DEGs, differentially expressed genes; DN-POLG, dominant-negative DNA polymerase gamma transgene; IPA, Ingenuity Pathway Analysis; mtDNA, mitochondrial DNA.
(XLSX)

S6 Data DEGs with differential DNA methylation in 143B rho0 cells. Δ%mCG, DNA methylation differences versus average of day 0; DEGs, differentially expressed genes.
(XLSX)

S7 Data Differentially expressed genes detected at day 9 of dox-inducible mtDNA depletion in NDI1/AOX cells by microarray (Affymetrix HG-U133 Plus 2.0). AOX, alternative oxidase; mtDNA, mitochondrial DNA; NDI1, nicotinamide adenine dinucleotide reduced dehydrogenase-like 1
(XLSX)

S8 Data Differentially expressed genes detected at day 9 of dox-inducible mtDNA depletion in DN-POLG cells by microarray (Affymetrix HG-U133 Plus 2.0). DN-POLG, dominant-negative DNA polymerase gamma transgene; mtDNA, mitochondrial DNA.
(XLSX)

S9 Data Original data from Fig 2F. (XLSX)

## Abbreviations

Δ%mCG: percentage of DNA methylation change
ΔΨm: mitochondrial membrane potential
1C: one carbon
2-HG: 2-hydroglutarate
5hmeC: 5-hydroxy-methyl-cytosine
5meC: 5-methyl-cytosine
acetyl-CoA: acetyl coenzyme A
AOX: alternative oxidase
ATF4: activating transcription factor 4
DEGs: differentially expressed genes
DNMT: DNA methyltransferase
DN-POLG: dominant-negative DNA polymerase gamma transgene
eNOS: endothelial nitric oxide synthase
ETC: electron transport chain
FADH_2_: flavin adenine dinucleotide hydroquinone
FDR: false discovery rate
FGF21: fibroblast growth factor 21
GABA: γ-aminobutyric acid
GDF15: growth differentiation factor 15
HEK293: human embryonic kidney 293
IPA: Ingenuity Pathway Analysis
KEGG: Kyoto Encyclopedia of Genes and Genomes
mtDNA: mitochondrial DNA
NADH: nicotinamide adenine dinucleotide reduced
NDI1: nicotinamide adenine dinucleotide reduced dehydrogenase-like 1
MFSD2A: major facilitator superfamily domain-containing protein 2a
MTA: 5-methyl-thioadenosine
mTOR: mammalian target of rapamycin
NIEHS: National Institute of Environmental Health Sciences
NIH: National Institute of Health
OR: odds ratio
PPARα: peroxisome proliferator activated receptor alpha
RNA-seq: RNA sequencing
ROS: reactive oxygen species
RPKM: reads per kilobase per million
SAH: S-adenosyl-homocysteine
SAM: S-adenosyl-methionine
SAT1: spermidine/spermine N-acetyl-transferase 1
TCA: tricarboxylic acid
TET: Ten-eleven translocation
Tet: tetracycline
TP53: tumor protein p53
