# Supplementary figures and images for: Mitochondrial nicotinamide adenine dinucleotide reduced (NADH) oxidation links the tricarboxylic acid (TCA) cycle with methionine metabolism and nuclear DNA methylation
Source: PLoS Biol. 2018 Apr 18;16(4):e2005707. doi: 10.1371/journal.pbio.2005707 (PMC5927466; doi:10.1371/journal.pbio.2005707)

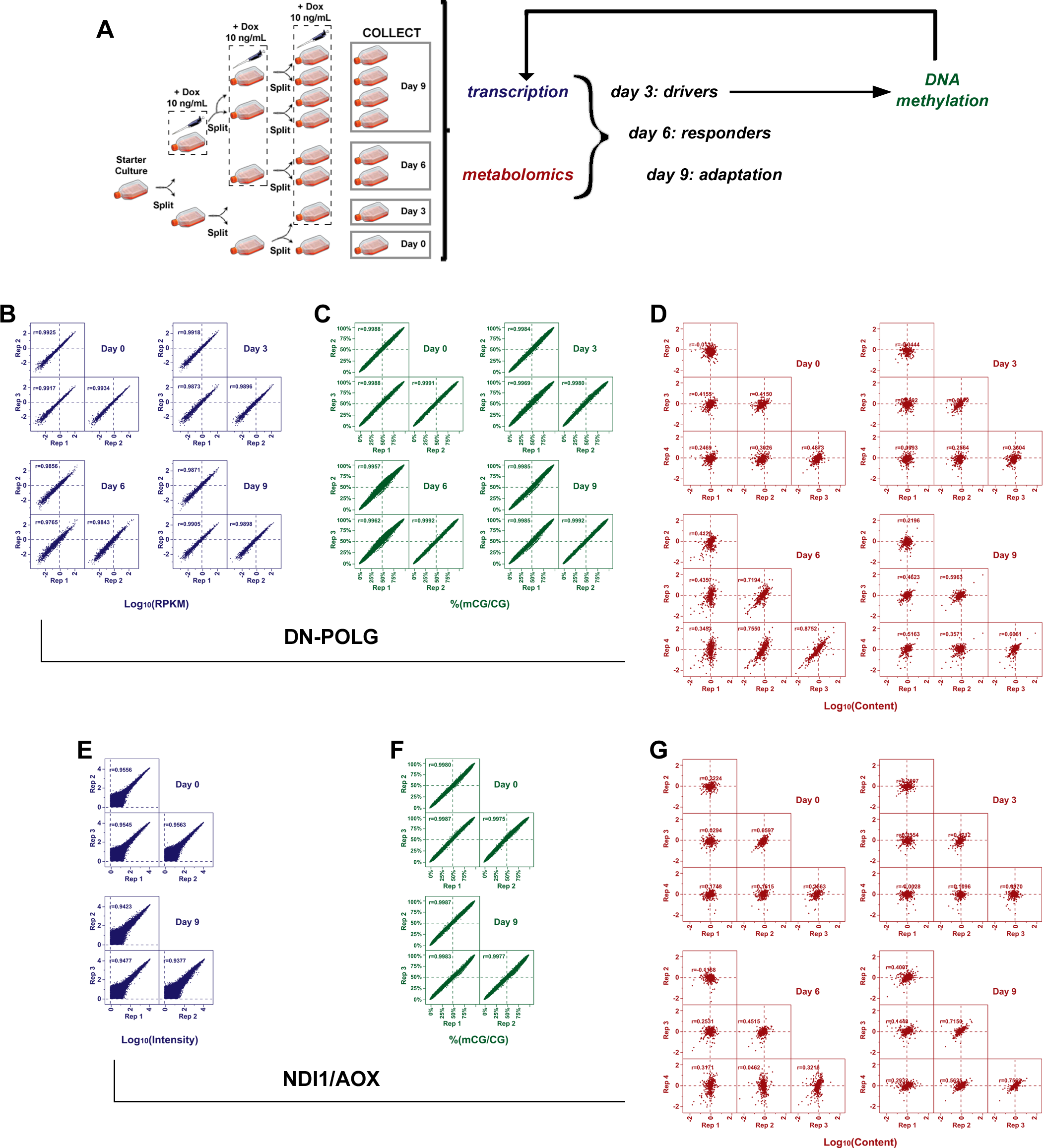

Supplement: S1 Fig — (A) Schematic representation of experimental procedure and data integration. Culture splits were performed every 3 days, corresponding to observed interval needed for noninduced DN-POLG or NDI1/AOX starter cultures to return to confluence after 1:2 subcultivation. As depicted, each cell culture round going from one single starter culture to individual cultures at day 0 (1 flask), 3 (1 flask), 6 (2 flasks, pooled), or 9 (4 flasks, pooled) of continuous supplementation with doxycycline (10 ng/mL) represents a single biological replicate. Independent biological replicates per cell model and per timepoint were produced for different assays: transcriptomics, N = 3; metabolomics, N = 4; DNA methylation, N = 3. (B–D) Reproducibility of biochemical output among DN-POLG biological replicates as shown by pairwise concordance and Pearson’s correlation r within different assays: (B) RNA-seq (Log10[RPKM], poly[A]-enriched RNA, uniquely aligned reads to hg19 reference genome; days 0, 3, 6, and 9); (C) Illumina HM-450K DNA Methylation BeadArrays (% mCG/CG, bisulfite-converted DNA; days 0, 3, 6, and 9); and (D) Metabolon mass spectrometry (Log10[Content], arbitrary units; days 0, 3, 6, and 9). (E–G) Reproducibility of biochemical output among NDI1/AOX biological replicates, as shown by pairwise concordance and Pearson’s correlation r within different assays: (E) Affymetrix HG-U133 Plus 2.0 Microarrays (Log10[Intensity], Total RNA; days 0 and 9); (F) Illumina HM-450K DNA Methylation BeadArrays (%mCG/CG, bisulfite-converted DNA; days 0 and 9); and (G) Metabolon mass spectrometry (Log10[Content], arbitrary units; days 0, 3, 6, and 9). Underlying data are reported in S1 Data for (B); S4 Data for (C) and (F); S3 Data for (D) and (G); and S7 Data for (E). %mCG, percentage of DNA methylation; AOX, alternative oxidase; DN-POLG, dominant-negative DNA polymerase gamma transgene; mtDNA, mitochondrial DNA; NDI1, nicotinamide adenine dinucleotide reduced dehydrogenase-like 1; RNA-seq, RNA seque [file pbio.2005707.s001.tif]

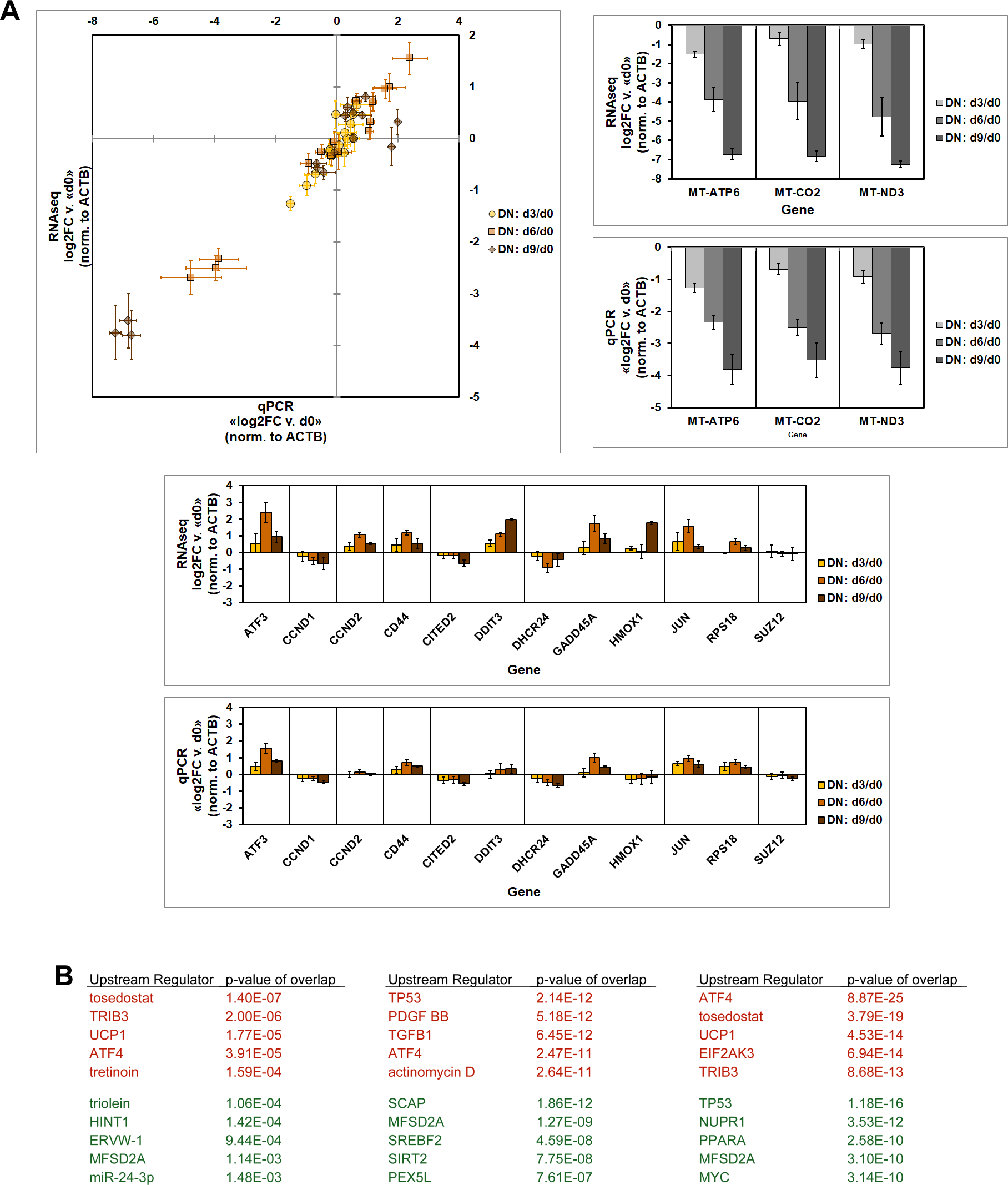

Supplement: S2 Fig — (A) Left upper panel depicts concordance of relative expression estimates normalized to housekeeping ACTB between RNA-seq and qPCR experiments for 12 nuclear-encoded DEGs and 3 mtDNA-encoded genes; increasingly darker shades of brown (nuclear-encoded) and gray (mtDNA-encoded) depict relative gene expression values at days 3, 6, and 9 each versus day 0. qPCR was performed from cDNA templates derived using the same total RNA extracts for both techniques (N = 3 per timepoint) and were performed in technical triplicates for each of the 3 biological replicates. Right top panels show graphs for mtDNA-encoded genes while the bottom panels depict data from nuclear DNA-encoded transcripts. Underlying data are reported in S1 Data. (B) Top 5 significantly enriched upstream regulators for upregulated and downregulated DEGs in DN-POLG cells at days 3, 6, and 9 of dox-inducible mtDNA depletion, per Ingenuity Pathway Analysis. ACTB, ß-actin; DEGs, differentially expressed genes; DN-POLG, dominant-negative DNA polymerase gamma transgene; mtDNA, mitochondrial DNA; qPCR, quantitative polymerase chain reaction; RNA-seq, RNA sequencing. (TIF) [file pbio.2005707.s002.tif]

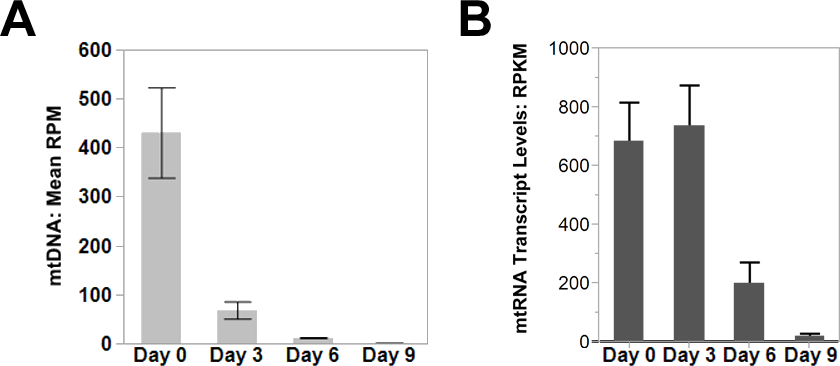

Supplement: S4 Fig — (A) Average normalized read counts (RPM; bar plots: mean ± SEM) of mtDNA fragments obtained by next-generation sequencing of whole-cell DNA for DN-POLG cells; N = 2 per timepoint. (B) Average normalized read counts (reads per kilobase per million reads, RPKM; bar plots: mean ± SEM) of mtDNA transcripts (mtRNA) obtained by RNA-seq for DN-POLG cells; N = 3 per timepoint. Underlying data are reported in S1 Data. DN-POLG, dominant-negative DNA polymerase gamma transgene; mtDNA, mitochondrial DNA; RNA-seq, RNA sequencing; RPKM, reads per kilobase per million reads; RPM, reads per million reads. (TIF) [file pbio.2005707.s004.tif]

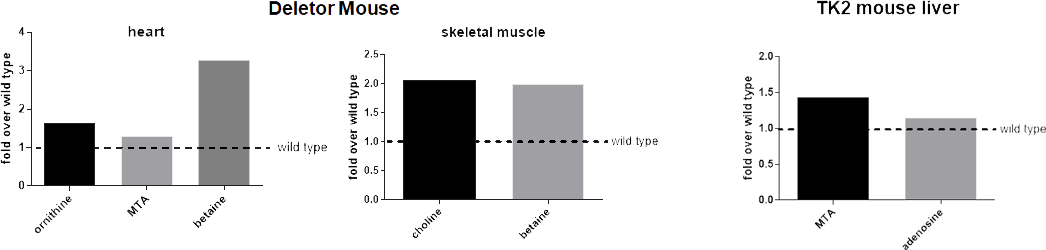

Supplement: S6 Fig — Graphs depict levels of metabolites in Deletor mice (skeletal muscle or heart) based on the fold-change relative to wild-type littermates, as reported in [9]. TK2 data were calculated based on normalized metabolite values from the KOs versus wild-type counterparts, as reported in [41]. DN-POLG, dominant-negative DNA polymerase gamma transgene; KO, knockout; MTA, 5-methyl-thioadenosine; mtDNA, mitochondrial DNA; TK2, timidine kinase 2 (TIF) [file pbio.2005707.s006.tif]
