# Supplementary material for: Mitochondrial nicotinamide adenine dinucleotide reduced (NADH) oxidation links the tricarboxylic acid (TCA) cycle with methionine metabolism and nuclear DNA methylation
Source: PLoS Biol. 2018 Apr 18;16(4):e2005707. doi: 10.1371/journal.pbio.2005707 (PMC5927466; doi:10.1371/journal.pbio.2005707)
Supplement: S3 Fig — Metabolomics was performed in DN-POLG cells at days 0, 3, 6, and 9; N = 4 per time point. Differentially enriched metabolites were identified based on log2-transformed fold-changes in arbitrary detection units versus the mean at day 0 by a two-way ANOVA test (metabolite × time) at an adjusted Benjamini-Hochberg p ≤ 0.05, and are detailed in S3 Data. (A) Number of differential metabolites identified in DN-POLG cells at days 3, 6, and 9 of doxycycline supplementation compared to day 0 (circle plots, top), and their overlap between time points (Venn diagram, bottom). (B) Heatmap of significantly represented canonical metabolic pathways per Ingenuity Pathway Analysis [−log(p) > 1.3] based on differentially enriched metabolites in DN-POLG cells at days 3, 6, and 9 of dox-inducible mtDNA depletion. Coloring of listed pathway names correspond to clades of pathways inferred by unsupervised clustering of enrichment scores across timepoints. (C–G) Data integration of average log2-fold changes relative to day 0 in gene expression (down: green, up: red; numerical values reported in S1 Data) and metabolite enrichment (down: blue, up: red; numerical values reported in S3 Data) with PathVisio engine for (C) nucleotide metabolism, (D) Methionine De Novo and Salvage Pathway, (E) One-Carbon Metabolism and Related Pathways, (F) Amino Acid Interconversion, and (G) Amino Acid Metabolism. (H) Depiction of three metabolic nodes identified as the main drivers of the response to mtDNA depletion in DN-POLG cells by day 3, per Ingenuity Pathway Analysis. acetyl-Coa, acetyl coenzyme A; DN-POLG, dominant-negative DNA polymerase gamma transgene; mtDNA, mitochondrial DNA; TCA, tricarboxylic acid. (PDF) [file pbio.2005707.s003.pdf]

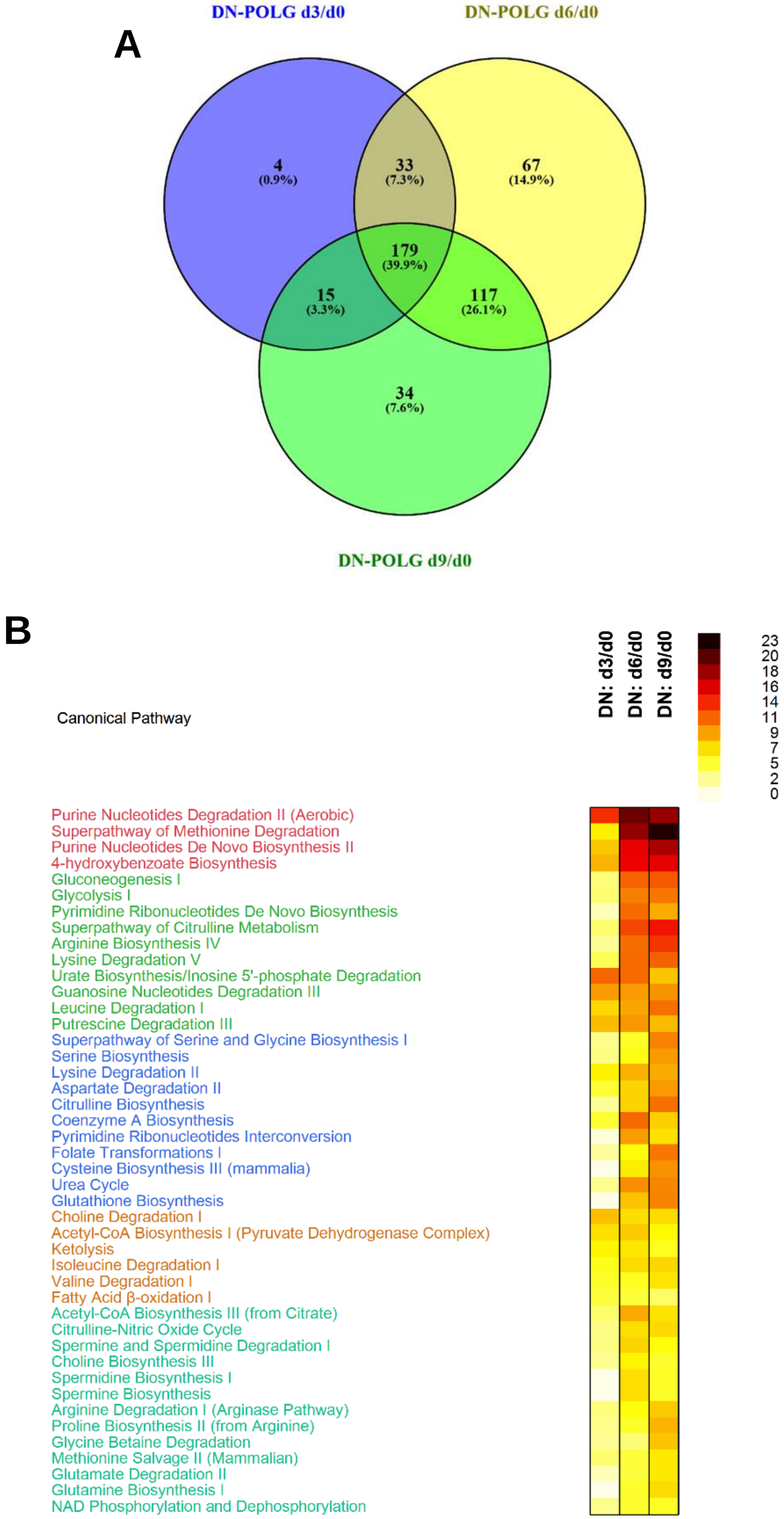
A
DN-POLG d3/d0
DN-POLG d6/d0
DN-POLG d9/d0
4
(0.9%)
33
(7.3%)
67
(14.9%)
179
(39.9%)
15
(3.3%)
117
(26.1%)
34
(7.6%)
B
Canonical Pathway
DN: d3/d0
DN: d6/d0
DN: d9/d0
23
20
18
16
14
11
9
7
5
2
0
Purine Nucleotides Degradation II (Aerobic)
Superpathway of Methionine Degradation
Purine Nucleotides De Novo Biosynthesis II
4-hydroxybenzoate Biosynthesis
Gluconeogenesis I
Glycolysis I
Pyrimidine Ribonucleotides De Novo Biosynthesis
Superpathway of Citrulline Metabolism
Arginine Biosynthesis IV
Lysine Degradation V
Urate Biosynthesis/Inosine 5'-phosphate Degradation
Guanosine Nucleotides Degradation III
Leucine Degradation I
Putrescine Degradation III
Superpathway of Serine and Glycine Biosynthesis I
Serine Biosynthesis
Lysine Degradation II
Aspartate Degradation II
Citrulline Biosynthesis
Coenzyme A Biosynthesis
Pyrimidine Ribonucleotides Interconversion
Folate Transformations I
Cysteine Biosynthesis III (mammalia)
Urea Cycle
Glutathione Biosynthesis
Choline Degradation I
Acetyl-CoA Biosynthesis I (Pyruvate Dehydrogenase Complex)
Ketolysis
Isoleucine Degradation I
Valine Degradation I
Fatty Acid β-oxidation I
Acetyl-CoA Biosynthesis III (from Citrate)
Citrulline-Nitric Oxide Cycle
Spermine and Spermidine Degradation I
Choline Biosynthesis III
Spermidine Biosynthesis I
Spermine Biosynthesis
Arginine Degradation I (Arginase Pathway)
Proline Biosynthesis II (from Arginine)
Glycine Betaine Degradation
Methionine Salvage II (Mammalian)
Glutamate Degradation II
Glutamine Biosynthesis I
NAD Phosphorylation and Dephosphorylation

C

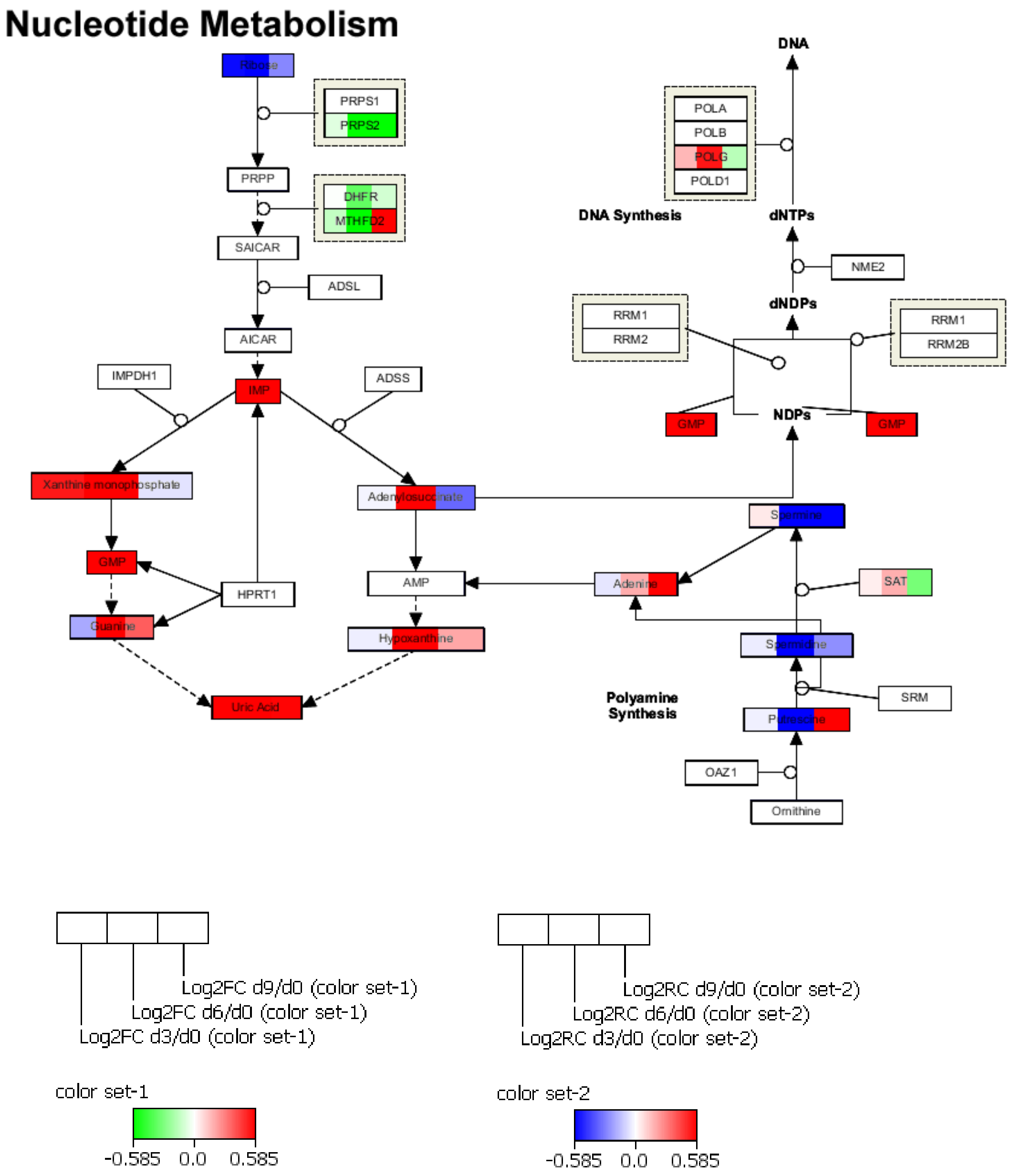
Nucleotide Metabolism
Ribose
PRPS1
PRPS2
PRPP
DHFR
MTHFD2
SAICAR
ADSL
AICAR
IMPDH1
IMP
ADSS
Xanthine monophosphate
Adenylosuccinate
GMP
HPRT1
AMP
Guanine
Hypoxanthine
Uric Acid
DNA
POLA
POLB
POLG
POLD1
DNA Synthesis
dNTPs
NME2
dNDPs
RRM1
RRM2
RRM1
RRM2B
GMP
NDPs
GMP
Spermine
SAT
Adenine
Spermidine
Polyamine Synthesis
SRM
Putrescine
OAZ1
Ornithine
Log2FC d9/d0 (color set-1)
Log2FC d6/d0 (color set-1)
Log2FC d3/d0 (color set-1)
color set-1
-0.585
0.0
0.585
Log2RC d9/d0 (color set-2)
Log2RC d6/d0 (color set-2)
Log2RC d3/d0 (color set-2)
color set-2
-0.585
0.0
0.585

D

Methionine De Novo and Salvage Pathway

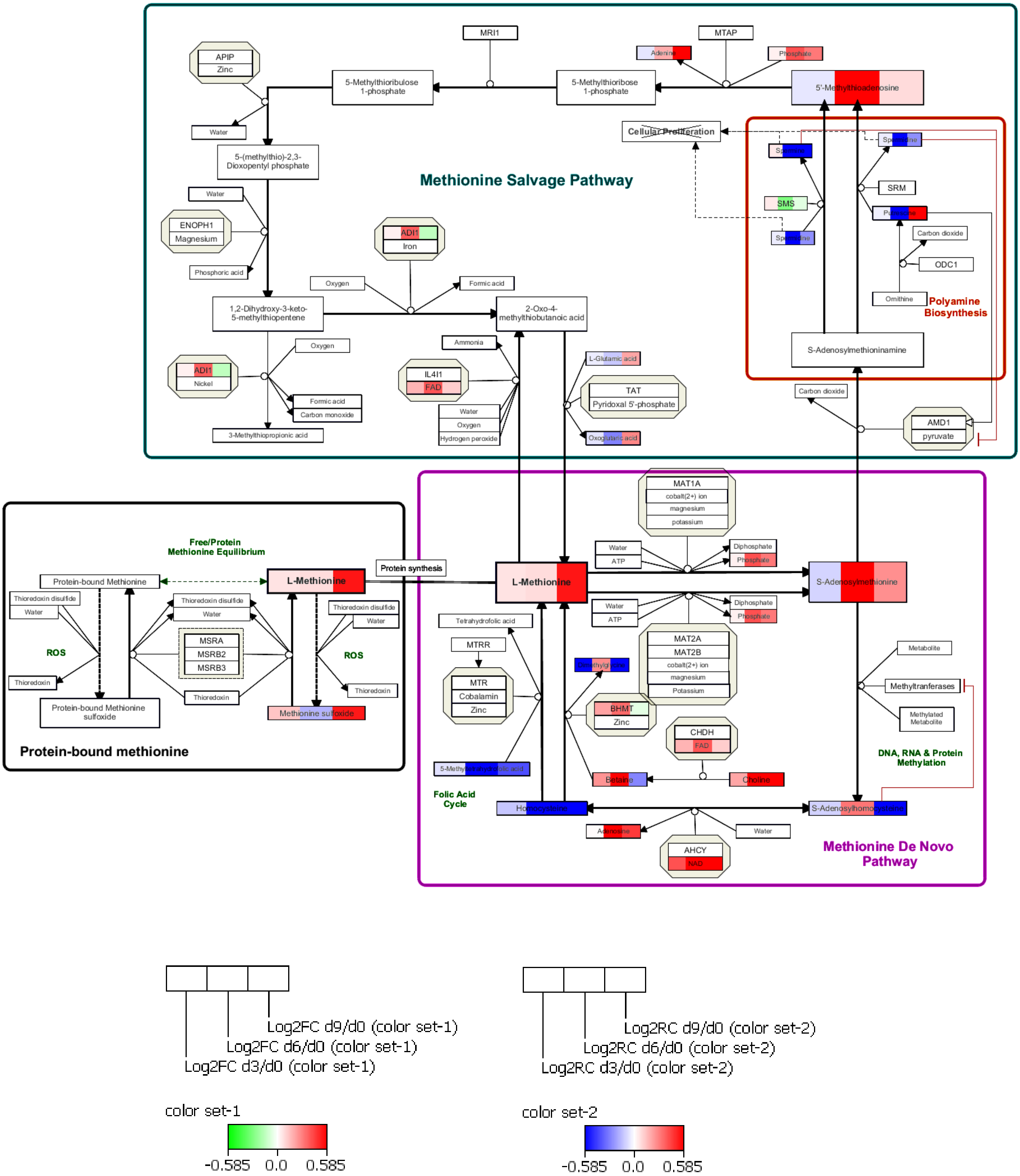

E

One-Carbon Metabolism and Related Pathways

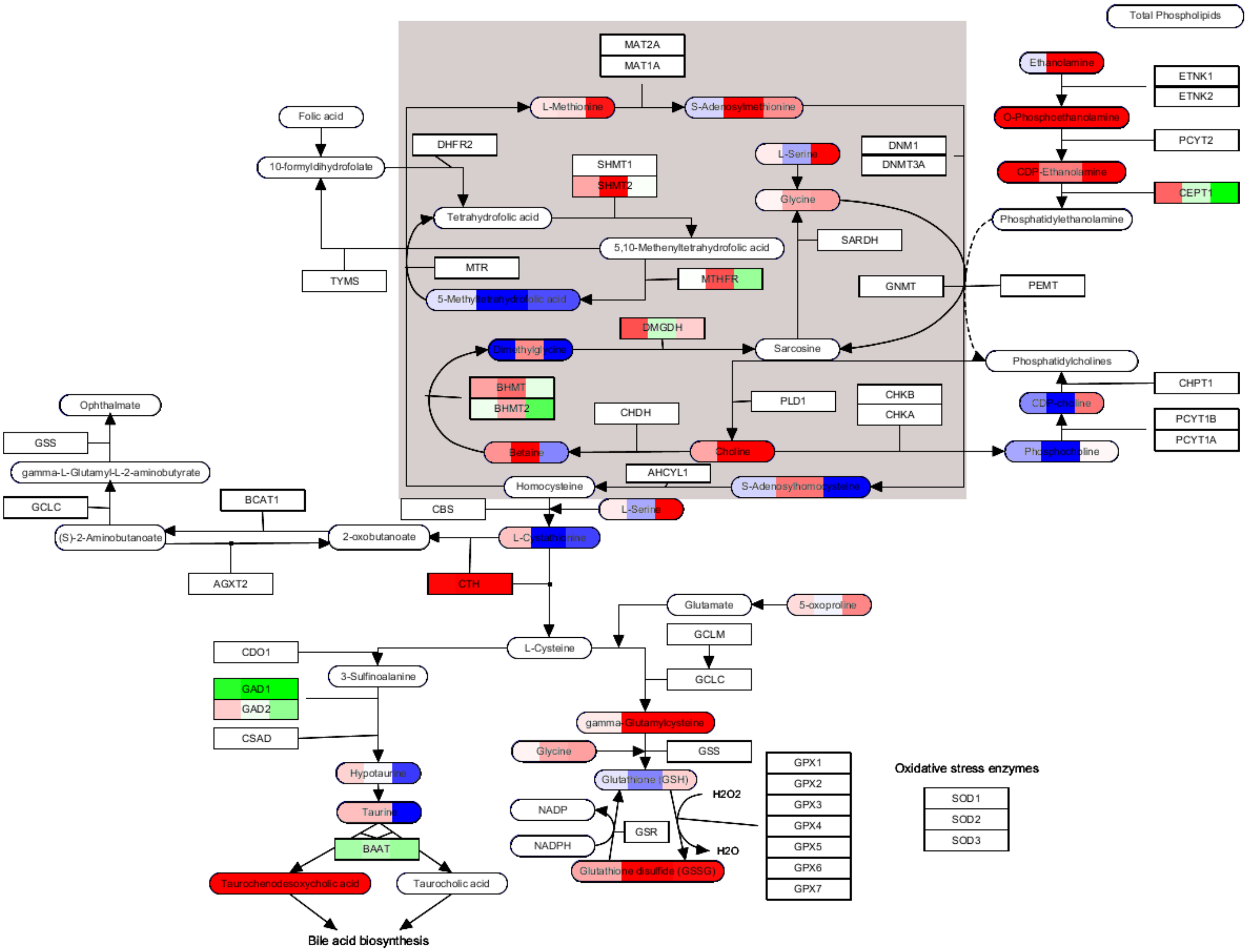

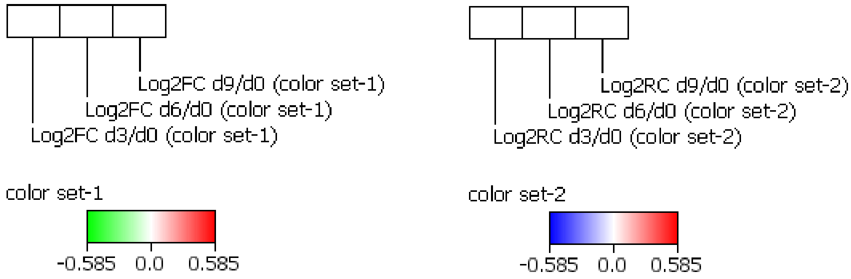

F

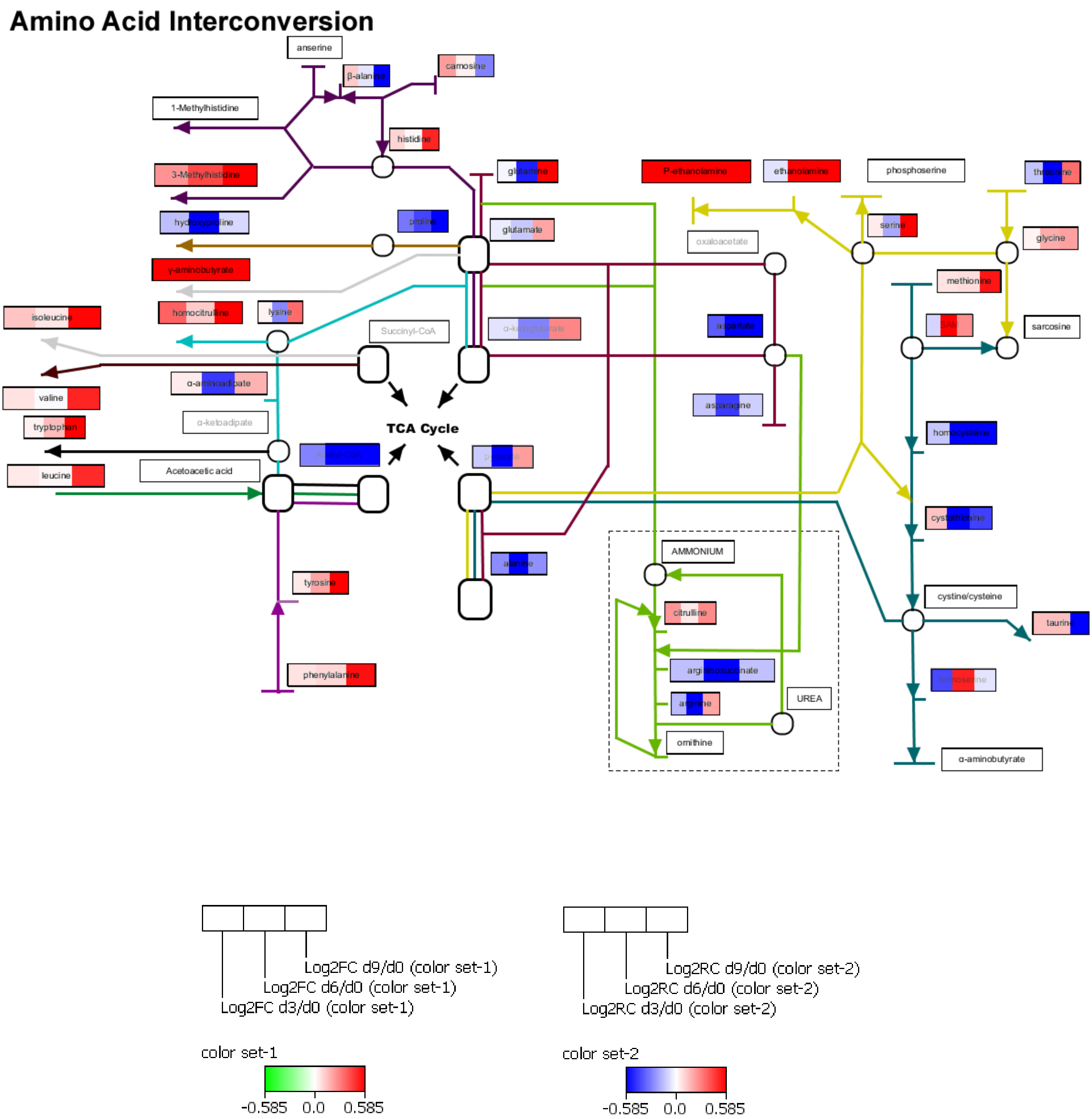

Amino Acid Interconversion
anserine
carnosine
β-alanine
1-Methylhistidine
histidine
3-Methylhistidine
glutamine
P-ethanolamine
ethanolamine
phosphoserine
threonine
hydroxyproline
proline
glutamate
serine
oxaloacetate
glycine
γ-aminobutyrate
methionine
isoleucine
homocitrulline
lysine
Succinyl-CoA
α-ketoglutarate
aspartate
SAM
sarcosine
α-aminoadipate
valine
asparagine
tryptophan
α-ketoadipate
TCA Cycle
homocysteine
leucine
Acetoacetic acid
pyruvate
cystathionine
AMMONIUM
alanine
tyrosine
citrulline
cystine/cysteine
taurine
argininosuccinate
phenylalanine
arginine
UREA
homoserine
ornithine
α-aminobutyrate
Log2FC d9/d0 (color set-1)
Log2FC d6/d0 (color set-1)
Log2FC d3/d0 (color set-1)
Log2RC d9/d0 (color set-2)
Log2RC d6/d0 (color set-2)
Log2RC d3/d0 (color set-2)
color set-1
-0.585 0.0 0.585
color set-2
-0.585 0.0 0.585

G

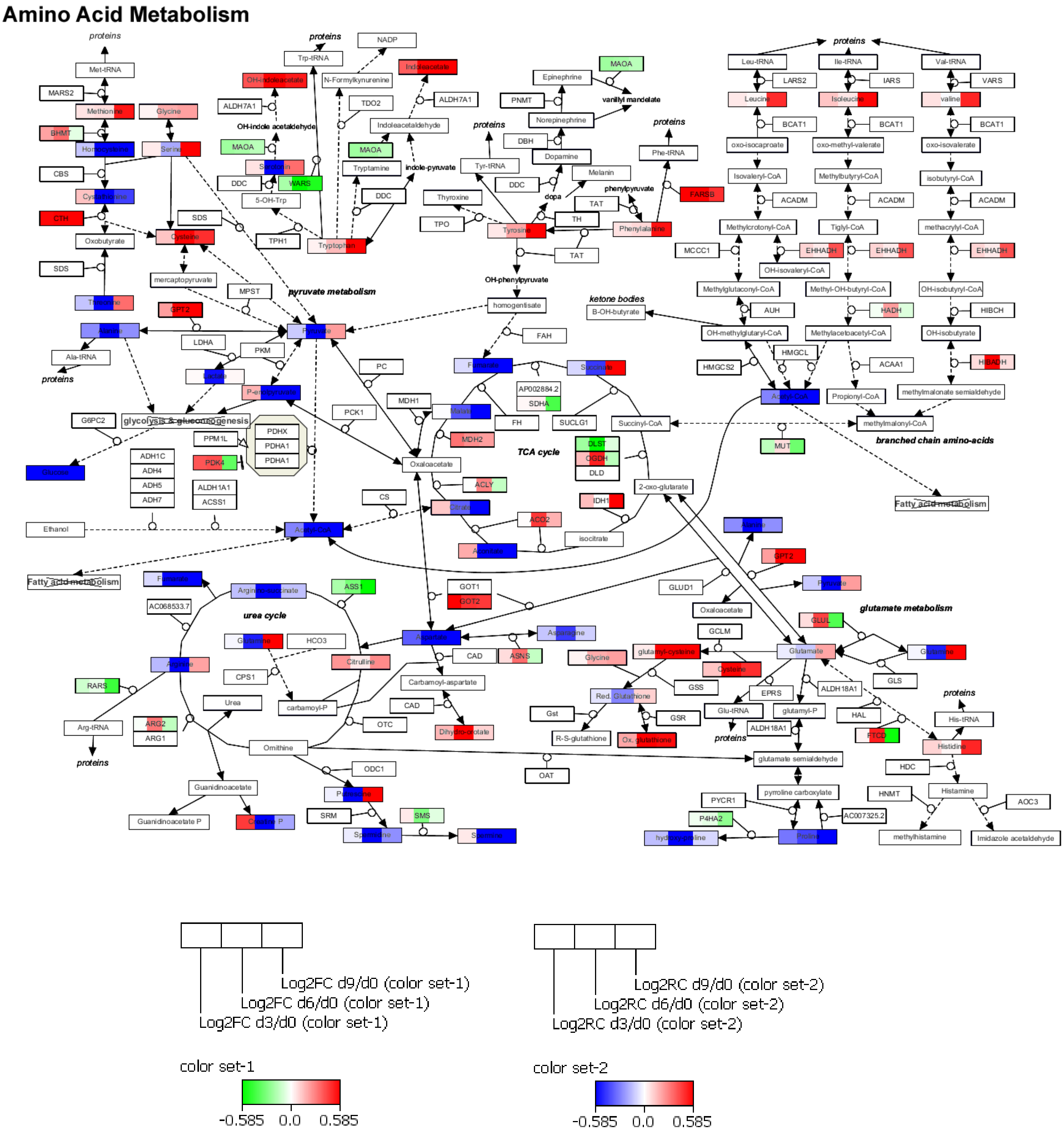
Amino Acid Metabolism
pyruvate metabolism
TCA cycle
ketone bodies
branched chain amino-acids
urea cycle
glutamate metabolism
glycolysis & gluconeogenesis
Fatty acid metabolism
proteins
Log2FC d9/d0 (color set-1)
Log2FC d6/d0 (color set-1)
Log2FC d3/d0 (color set-1)
Log2RC d9/d0 (color set-2)
Log2RC d6/d0 (color set-2)
Log2RC d3/d0 (color set-2)
color set-1
-0.585 0.0 0.585
color set-2
-0.585 0.0 0.585

H

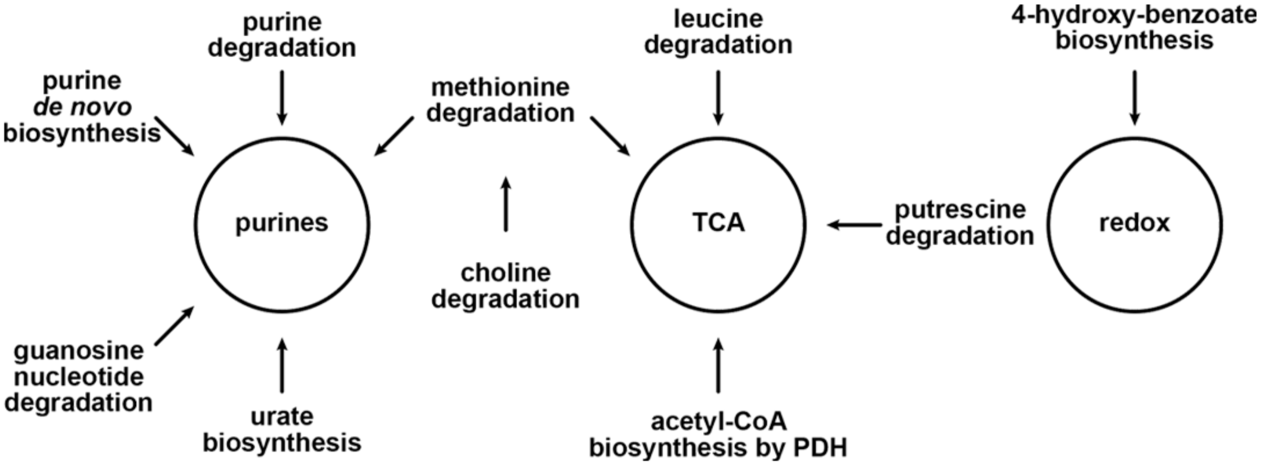
purine
degradation
purine
de novo
biosynthesis
methionine
degradation
leucine
degradation
4-hydroxy-benzoate
biosynthesis
purines
TCA
redox
choline
degradation
putrescine
degradation
guanosine
nucleotide
degradation
urate
biosynthesis
acetyl-CoA
biosynthesis by PDH
