# Supplementary material for: Mitochondrial nicotinamide adenine dinucleotide reduced (NADH) oxidation links the tricarboxylic acid (TCA) cycle with methionine metabolism and nuclear DNA methylation
Source: PLoS Biol. 2018 Apr 18;16(4):e2005707. doi: 10.1371/journal.pbio.2005707 (PMC5927466; doi:10.1371/journal.pbio.2005707)
Supplement: S5 Fig — (A) Violin plots depict Δ%mCG for DML observed in DN-POLG cells by HM-450K BeadArrays broken down by probe annotated location: IGR, TSS 1500 and TSS 200, 5'UTR, 1st exon (exon 1 as per annotated reference genome), body (gene body), and 3'UTR. Number of DML at each time point is shown at the top of each plot frame within each time point and probe location class. (B) Unsupervised hierarchical clustering showing the percentage of methylation of individual locus, presented in each row, in either rho+ or rho0 cells based on Illumina 450K methylation arrays; N = 3. (C) Immunofluorescence using antibodies again 5meC or 5hmeC in both cell types; images taken with confocal microscope; blue—DAPI-stained nucleus; controls containing only primary or only secondary antibodies or background fluorescence are omitted. (D) Dot blots probing levels of 5meC or 5hmeC were performed in technical triplicates; N = 12. (E) Number of DMEGs in DN-POLG cells at each time point is shown above each panel. All identified differentially methylated probes relative to day 0 associated with DEGs in DN-POLG cells are shown. X-axis depicts the degree of methylation change while the y-axis shows the expression change for each respective DEG. (F) Expected versus observed number of genes that bear or do not bear changes in DNA methylation relative to DEGs in DN-POLG cells; p-value was calculated using chi-squared test. (G) Bar graph shows metabolic pathways identified enriched in 143B rho0 cells chronically depleted of mtDNA based on 621 DEGs that were also differentially methylated, per Ingenuity Pathway Analysis; the top x-axis depicts log10-tranformed significance score of enriched pathways, whereas the bottom ratio x-axis (bottom) and line plot (orange) reflects the proportion of members within each pathway that are present in the dataset. (H) Concordance between gene expression directionality (y-axis) and change in DNA methylation levels (x-axis) are presented for each time point. The number of DEGs [file pbio.2005707.s005.pdf]

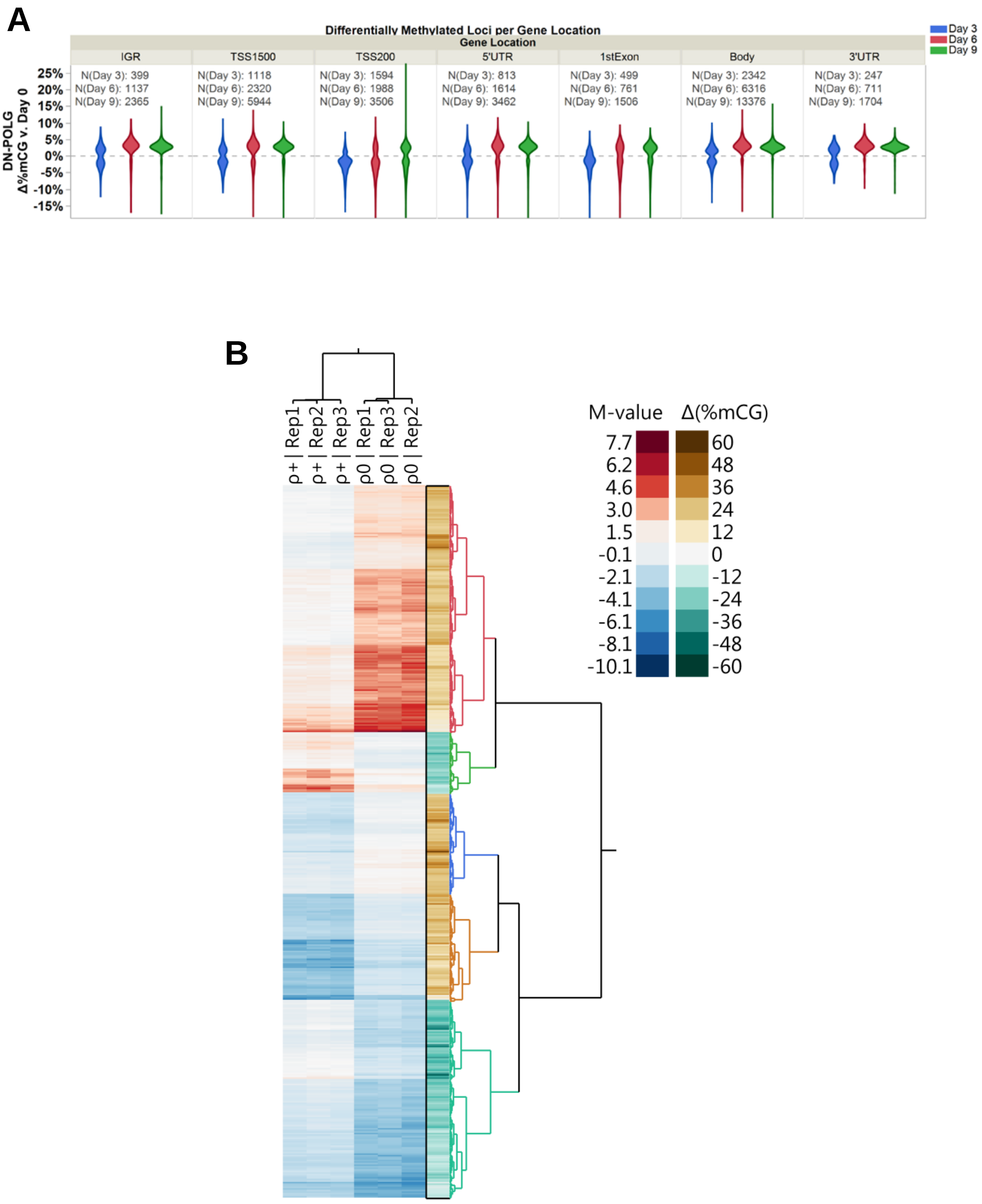
A
Differentially Methylated Loci per Gene Location
Gene Location
IGR
TSS1500
TSS200
5'UTR
1stExon
Body
3'UTR
Day 3
Day 6
Day 9
DN-POLG
Δ%mCG v. Day 0
25%
20%
15%
10%
5%
0%
-5%
-10%
-15%
N(Day 3): 399
N(Day 6): 1137
N(Day 9): 2365
N(Day 3): 1118
N(Day 6): 2320
N(Day 9): 5944
N(Day 3): 1594
N(Day 6): 1988
N(Day 9): 3506
N(Day 3): 813
N(Day 6): 1614
N(Day 9): 3462
N(Day 3): 499
N(Day 6): 761
N(Day 9): 1506
N(Day 3): 2342
N(Day 6): 6316
N(Day 9): 13376
N(Day 3): 247
N(Day 6): 711
N(Day 9): 1704
B
ρ+ | Rep1
ρ+ | Rep2
ρ+ | Rep3
ρ0 | Rep1
ρ0 | Rep3
ρ0 | Rep2
M-value
7.7
6.2
4.6
3.0
1.5
-0.1
-2.1
-4.1
-6.1
-8.1
-10.1
Δ(%mCG)
60
48
36
24
12
0
-12
-24
-36
-48
-60

C

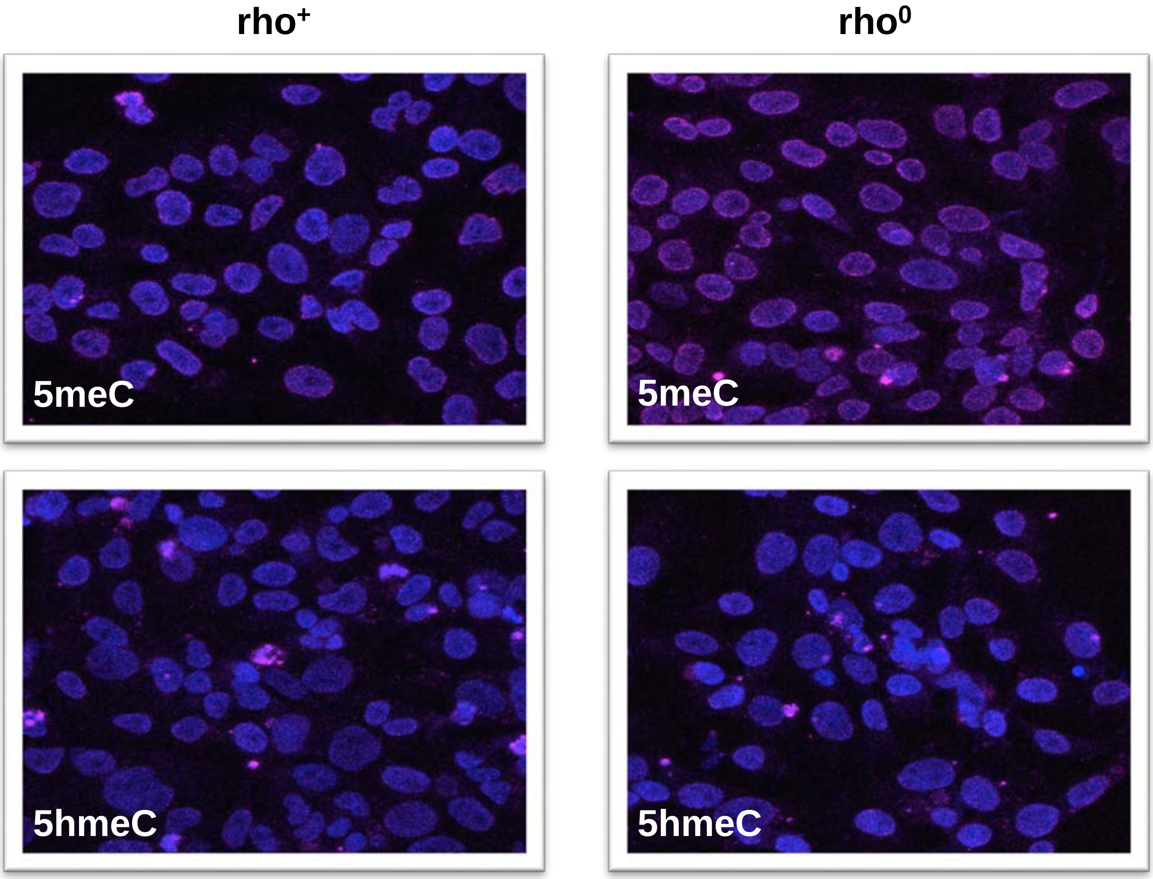
rho+
rho0
5meC
5meC
5hmeC
5hmeC

D

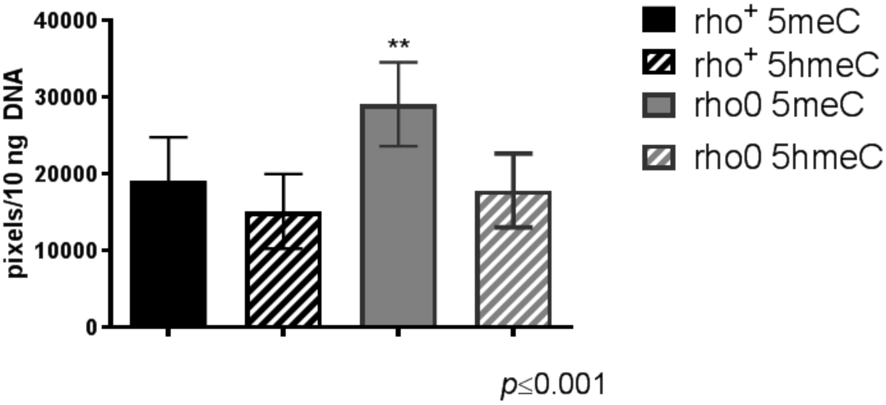
40000
30000
20000
10000
0
pixels/10 ng DNA
**
rho+ 5meC
rho+ 5hmeC
rho0 5meC
rho0 5hmeC
p≤0.001

E

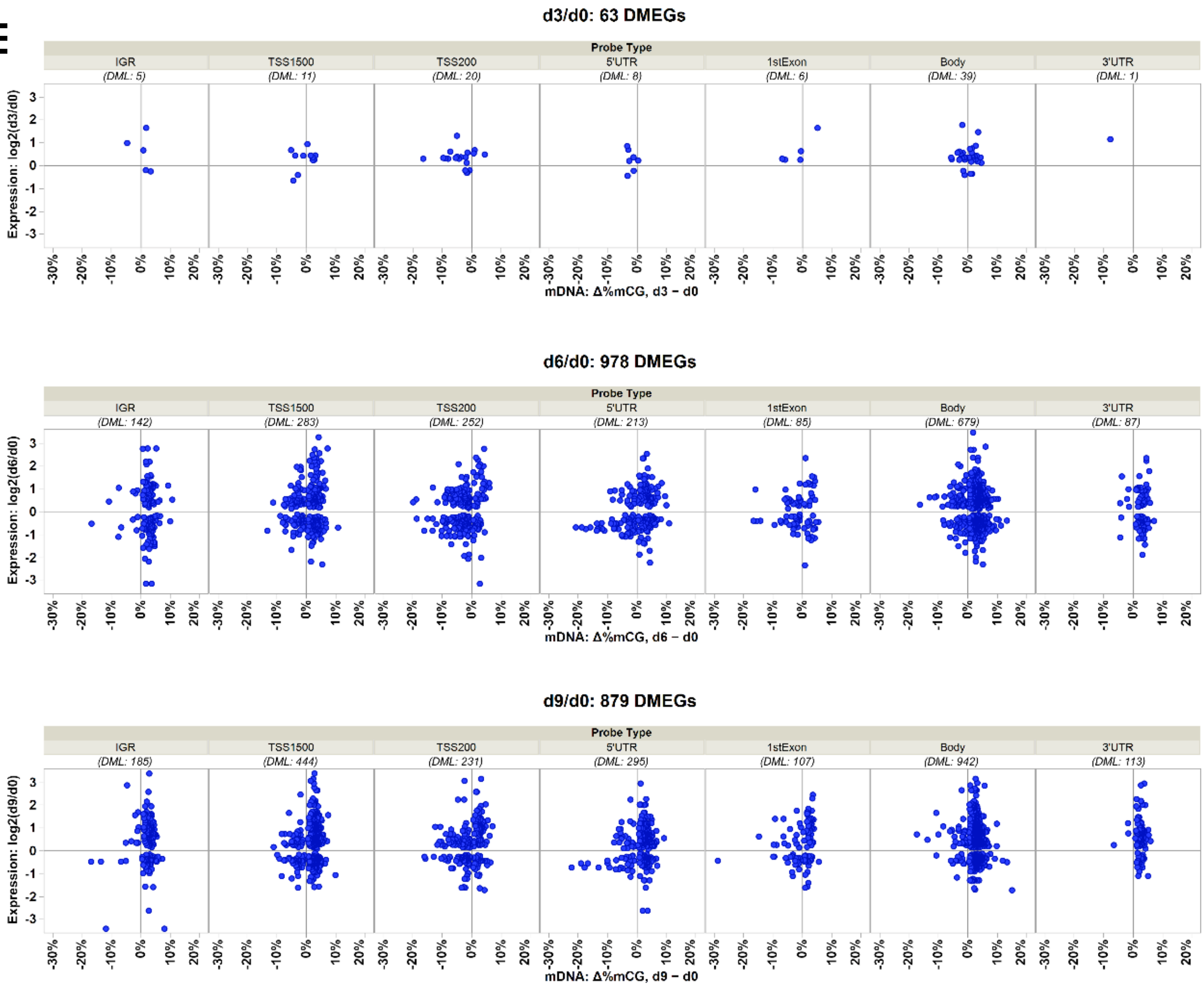

F

**DN-POLG: Total DEGs**	**2,850**
**DN-POLG: Total DMGs**	**12,461**
**P-value (Chi-sq)**	**4.0E-07**
**Odds Ratio (O/E)**	**0.81**

	Observed		Expected		
	DMG	Not DMG	DMG	Not DMG	Rate
**DEG**	1,627	1,223	1,749.1	1,100.9	57.09%
**Not DEG**	10,834	6,620	10,711.9	6,742.1	62.07%

G

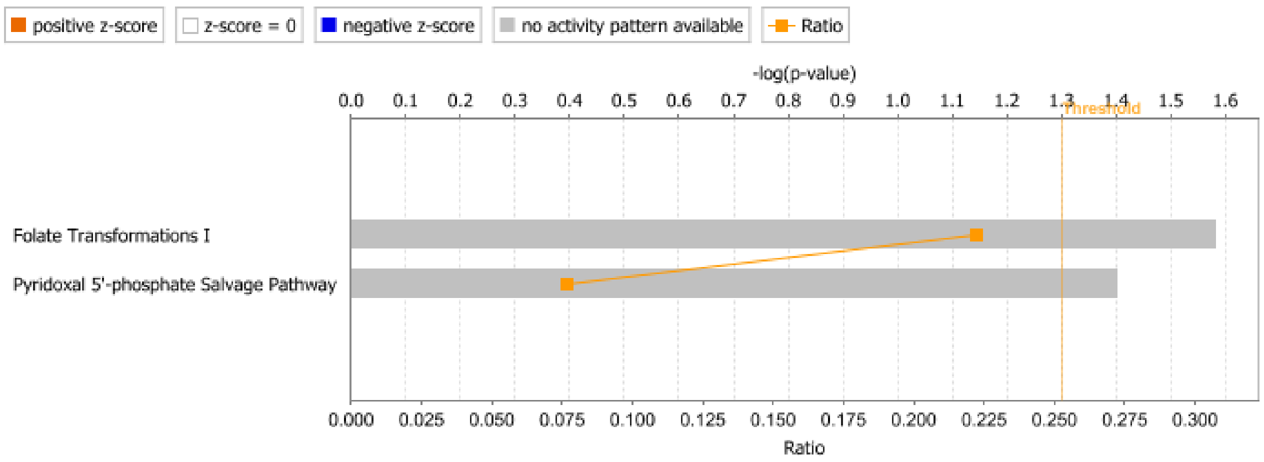

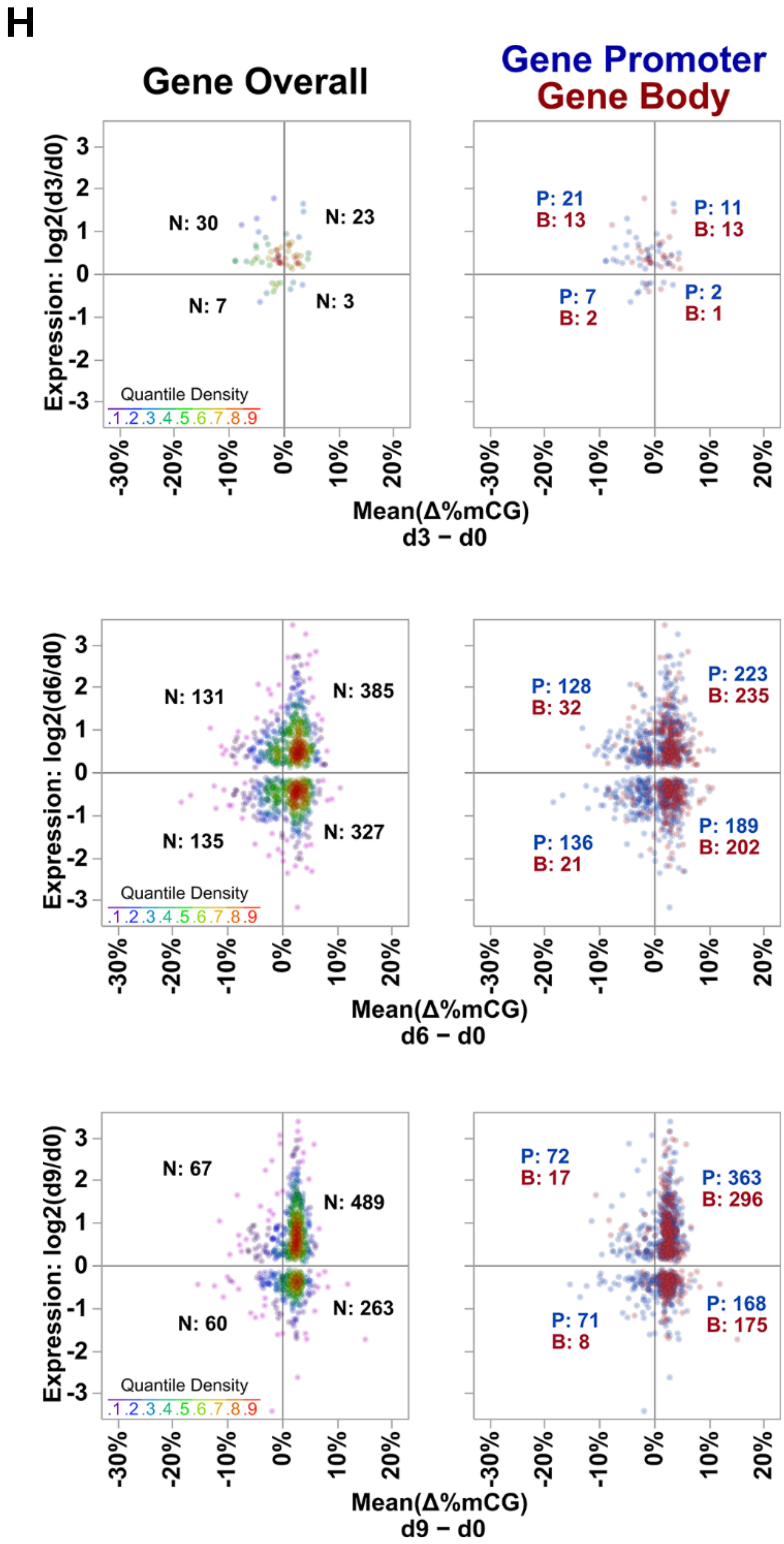
H
Gene Overall
Gene Promoter
Gene Body
Expression: log2(d3/d0)
N: 30
N: 23
N: 7
N: 3
P: 21
B: 13
P: 11
B: 13
P: 7
B: 2
P: 2
B: 1
Quantile Density
.1 .2 .3 .4 .5 .6 .7 .8 .9
-30%
-20%
-10%
0%
10%
20%
Mean(Δ%mCG)
d3 − d0
Expression: log2(d6/d0)
N: 131
N: 385
N: 135
N: 327
P: 128
B: 32
P: 223
B: 235
P: 136
B: 21
P: 189
B: 202
Mean(Δ%mCG)
d6 − d0
Expression: log2(d9/d0)
N: 67
N: 489
N: 60
N: 263
P: 72
B: 17
P: 363
B: 296
P: 71
B: 8
P: 168
B: 175
Mean(Δ%mCG)
d9 − d0
